# Establishing normal metabolism and differentiation in hepatocellular carcinoma cells by culturing in adult human serum

**DOI:** 10.1038/s41598-018-29763-2

**Published:** 2018-08-03

**Authors:** Rineke Steenbergen, Martin Oti, Rob ter Horst, Wilson Tat, Chris Neufeldt, Alexandr Belovodskiy, Tiing Tiing Chua, Woo Jung Cho, Michael Joyce, Bas E. Dutilh, D. Lorne Tyrrell

**Affiliations:** 1 Li Ka Shing Institute of Virology, Dept. of Medical Microbiology and Immunology, University of Alberta, Edmonton, Canada; 2 Carlos Chagas Filho Biophysics Institute (IBCCF), Federal University of Rio de Janeiro (UFRJ), Rio de Janeiro, Brazil; 3 Centre for Molecular and Biomolecular Informatics, Radboud Institute for Molecular Life Sciences, Radboud University Nijmegen Medical Centre, Nijmegen, The Netherlands; 4 Theoretical Biology and Bioinformatics, Utrecht University, Utrecht, The Netherlands

## Abstract

Tissue culture medium routinely contains fetal bovine serum (FBS). Here we show that culturing human hepatoma cells in their native, adult serum (human serum, HS) results in the restoration of key morphological and metabolic features of normal liver cells. When moved to HS, these cells show differential transcription of 22–32% of the genes, stop proliferating, and assume a hepatocyte-like morphology. Metabolic analysis shows that the Warburg-like metabolic profile, typical for FBS-cultured cells, is replaced by a diverse metabolic profile consistent with *in vivo* hepatocytes, including the formation of large lipid and glycogen stores, increased glycogenesis, increased beta-oxidation and ketogenesis, and decreased glycolysis. Finally, organ-specific functions are restored, including xenobiotics degradation and secretion of bile, VLDL and albumin. Thus, organ-specific functions are not necessarily lost in cell cultures, but might be merely suppressed in FBS. The effect of serum is often overseen in cell culture and we provide a detailed study in the changes that occur and provide insight in some of the serum components that may play a role in the establishment of the differentiated phenotype.

## Introduction

Cancer cell lines are commonly used as a model to study physiological processes *in vitro*, because they are readily manipulated and can be cultured in large quantities. However, key morphological and metabolic features are often repressed or absent in rapidly dividing cells. Hepatocellular carcinoma (HCC) cell lines lack key liver features, including cell polarization, VLDL secretion, and detoxification of xenobiotics^1,2^. Additionally, cancer cells, including hepatoma cells, typically have a cancer metabolic profile^3^, using aerobic glycolysis (‘the Warburg effect’) and glutaminolyis for energy production, which is not representative of normal liver physiology and metabolism, and of the regulatory role that healthy hepatocytes play in lipid and glucose homeostasis. Although cultured primary hepatocytes are more representative of hepatocytes *in vivo* than HCC cells, they are also expensive, only available in small quantities, and difficult to manipulate (e.g. CRISPR editing). Recently we demonstrated that choosing and alternative serum source alone can have major implications on cell functions: when HCC cells are cultured in their native adult serum undergo contact inhibition, and differentiate into a hepatocyte-like cell^4^. We showed that in these cells key hepatic functions, like VLDL secretion are restored, and for example the production of hepatitis C virus in differentiated cells increases more than a 1000-fold, while also producing HCV particles that are more representative of the particles that are circulating in the serum of HCV infected patients. HS cultured Huh7.5 cells, infected or not, can be maintained in HS for at least 100 days, without the need for sub-culturing. In the current study, we further investigated the cellular changes that occur in HCC cells that are cultured in HS instead of FBS. We used a combination of microarray analysis, microscopic techniques, and biological assays to show that the limitations of standard HCC cultures can be overcome by changing the serum. By replacing FBS with HS in the cell culture medium, Huh7.5 cells (i) become growth arrested, obtain an epithelial, cuboid morphology and become polarized; (ii) undergo complete metabolic reprogramming, with a reversal of the cancer metabolic profile (Warburg effect and glutaminolyis); (iii) diversify other metabolic pathways, with a reduction in glycolysis, an increase in glycogen storage (glycogenesis) and higher reliance on β-oxidation; and (iv) increase mRNAs of many CypP450 enzymes and CypP450 metabolic rates and increase or restore secretory processes, like VLDL, albumin and bile secretion.

Summarizing, we show that by simply placing cells in their native adult serum, extensive reprogramming of Huh7.5 can take place, and the morphology and functions that were considered lost in cancer cell lines can be restored. We discuss the relevance of these findings for *in vitro* research, given the central role metabolism plays in various physiological processes.

## Results

### Polarization, cytoskeletal organization and other morphological changes

**Figure 1:**
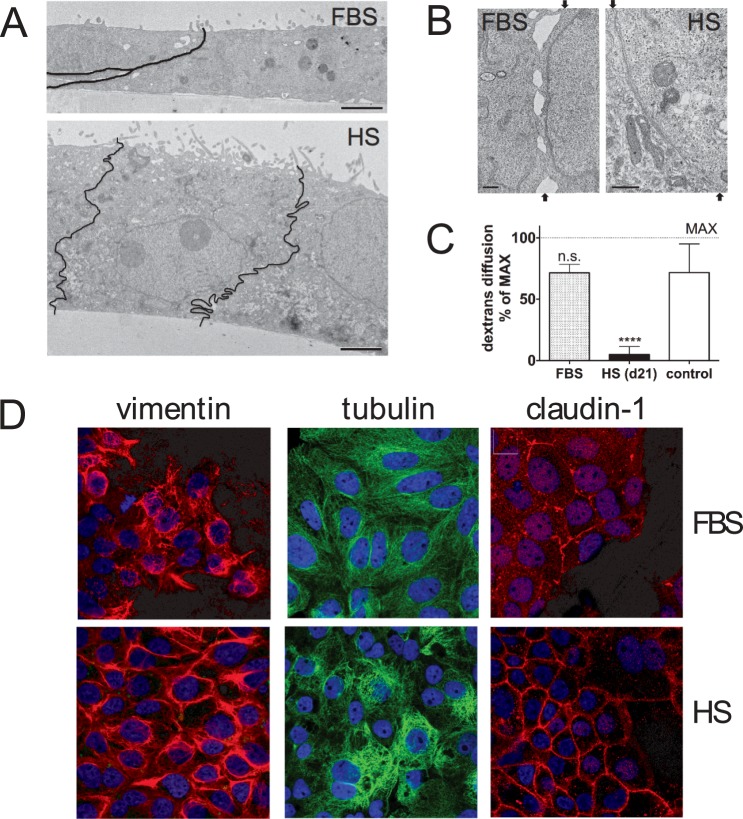
Morphology of Huh7.5 cells cultured in FBS- and HS-containing media. (**A**) Electron micrographs of sagittal sections of Huh7.5 cells that were cultured in FBS-containing media (top image) and HS-containing media (bottom image). Black lines indicate the location of the borders between two HS cells. The images were taken at the same magnification (bar is 2 µm). Shown is a representative figure from 2 experiments with 2 transwell dishes each. (**B**) Electron micrographs of the border between two adjacent cell in FBS (left) and in HS (right). The arrows indicate the start and end of the border region on the image. Shown is a representative image of 3 experiments with 2 dishes each. (**C**) Dextran diffusion rate across confluent layers of FBS (FBS) and HS-cultured cells (HS d21), grown on transwell dishes. Subconfluent cultures were used as a control (control). Data are normalized to maximal diffusion rate (MAX), measured on dishes without cells. Data are presented as mean with standard deviation, from 4 independent experiments with two transwell dishes each. (**D**) Confocal imaging of FBS and HS-cultured Huh7.5 cells. Cytoskeleton components vimentin and tubulin where stained, as well as claudin-1, a tight junction component. Images are representative images taken from 3 separate coverslips.

We investigated the effect of replacing FBS by HS in tissue culture media, on cell morphology and the gene expression profile of the HCC cell line Huh7.5. We first examined overall morphological changes resulting from extended culturing in HS. HS and FBS-cultured cells where grown on transwell dishes, prepared for electron microscopy and sectioned perpendicular to the membrane surface, so that a ‘side view’ of the cell is created (Fig. 1A). HS-cultured cells become cuboid, consistent with the *in vivo* hepatocyte phenotype. Moreover, their apical surface has a more pronounced epithelial character than FBS-cultured cells, with more and larger villi. HS-cultured cells are also tightly interconnected, with no open space in between, unlike their FBS-cultured counterparts. This is confirmed in higher magnification images of the cell boundaries (Fig. 1B). Increased cytoplasm density and altered organelle organization were also noted in HS-cultured cells as further described in Supplemental Data 1.

Cytoskeletal organization plays an important role in establishing polarization and cell shape. Therefore, we visualized cytoskeletal reorganization of tubulin, a microfilament, and vimentin, an intermediate filament, by confocal microscopy. Whereas vimentin is disorganized in FBS-cultured cells, a structured organization is seen in HS-cultured cells (d21). Tubulin appears more condensed in HS-cultured cells than in FBS-cultured cells, where staining is faint and more dispersed.

We also visualized the organization of claudin-1, a major component of the tight junction complex (Fig. 1D). Claudin-1 is present in FBS, but the staining is patchy, whereas in HS-cultured cells claudin-1 is distributed evenly around the entire cell, pointing at better barrier function. To test if such a barrier exists, we measured the diffusion rate of fluorescently labeled 70 kDa dextran conjugates across confluent layers of FBS-cultured cells or HS-cultured cells grown on transwell dishes. HS-cultured cells were almost impermeable to these conjugates, showing that a barrier is indeed established, whereas FBS monolayers remained permeable (Fig. 1C).

Concluding, morphological hallmarks of hepatocytes, including the formation of polarized cell layers consisting of tightly interconnected cuboid cells, can be achieved in Huh7.5 cells simply by culturing them in HS instead of FBS.

### Gene expression changes

**Figure 2:**
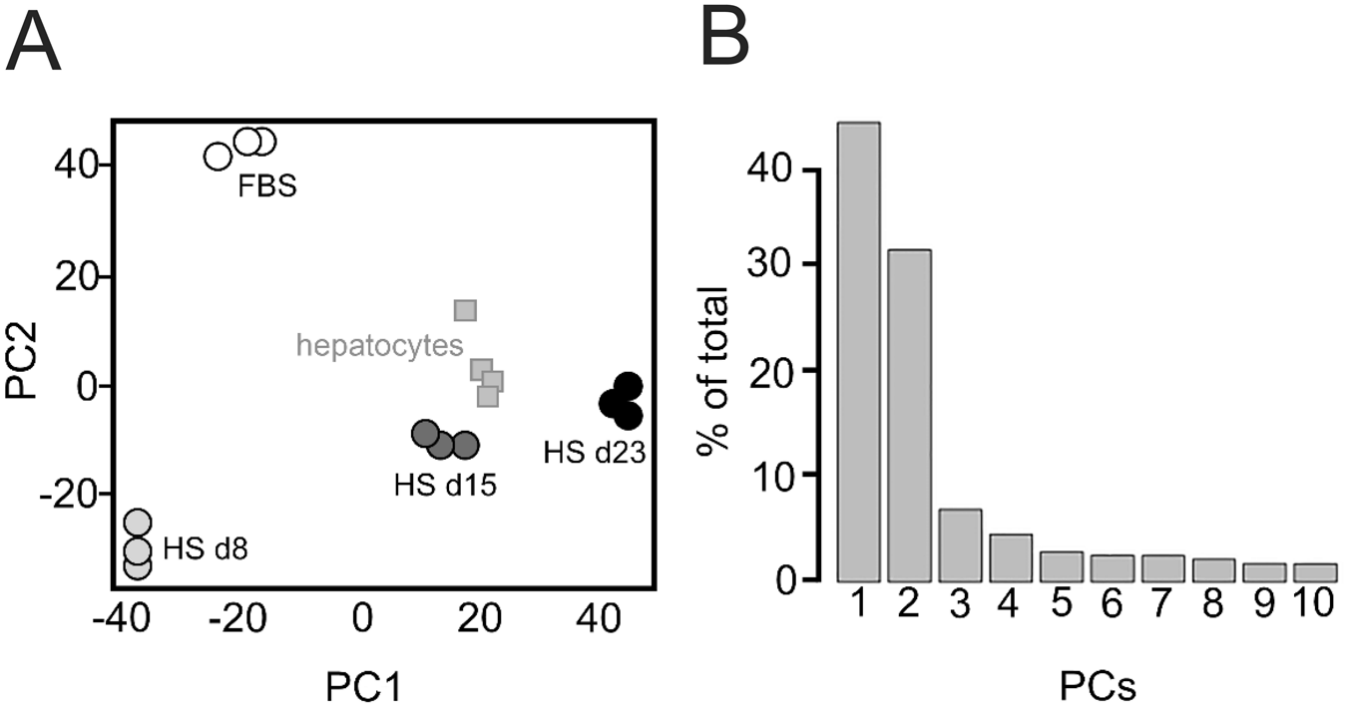
Principal component analysis. Principal component analysis (PCA) is a statistical method that is typically used as an exploratory method for data analysis, as it provides insight in the quality of a dataset, and in differences between experimental groups. (**A**) First two principal components (PCs) of triplicate genome-wide expression profiles of Huh7.5 cells in FBS and 8, 15, and 23 days after transfer to HS. To determine the similarity in gene expression between HS-cultured cells and primary hepatocytes, published hepatocyte expression profiles were projected onto the PCA, by applying the gene weights associated with PC1 and PC2 to the gene expression levels (squares). (**B**) Scree-plot indicating the total variance explained by the individual PCs. 75% of variance in gene expression is explained by the first two PCs principal components.

Next, we used genome-wide expression arrays to investigate the overall gene expression changes of cells cultured in HS for 8, 15 and 23 days compared to cells cultured in FBS. These time points were chosen because^4^: (i) HS-cultured cells become growth arrested after 7–10 days, (ii) around day 15 the first morphological signs of differentiation become apparent and (iii) after 21 days the differentiation process appears complete. Indeed, gene expression changed significantly (p < 0.05 after multiple testing correction) in 32% of the genes by day 23 (16,000 of 49,000 probes), revealing a complete cellular reprogramming upon shifting to HS media (Supplemental Data 2). Principal component analysis (PCA, Fig. 2A) showed a good experimental replicability, and a clear change in expression profile upon shifting the cells from FBS to HS. To determine the similarity in gene expression between HS-cultured cells and primary hepatocytes, published hepatocyte expression profiles were projected onto the PCA, by applying the gene weights associated with PC1 and PC2 to the gene expression levels. This showed close proximity of primary hepatocytes to the later stages of the HS-cultured HCC cells, suggesting differentiation towards a hepatocyte phenotype (Fig. 2).

### PAM clustering and DREM analysis

**Figure 3:**
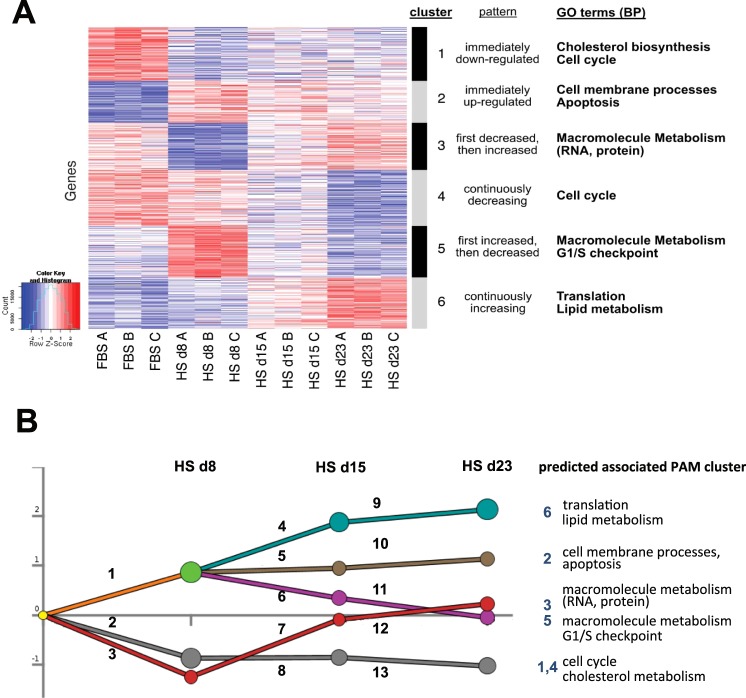
PAM clustering and DREM analysis. (**A**) PAM clustering (partitioning around medoids) of the microarray data into 6 clusters. Samples are on the x-axis while genes are on the y-axis. Gene expression values were standardized across the samples into Z-scores. Red indicates higher expression relative to other samples, blue indicates lower expression. The Gene Ontologies terms/Biological Processes (GO terms/BP) associated with the clusters were determined (Supplemental Data 4) and summarized on the right of the figure. (**B**) DREM Analysis. The Dynamic Regulatory Events Miner (DREM) allows one to model, analyze, and visualize transcriptional gene regulation dynamics. The method of DREM takes as input time series gene expression data and known transcription factor-gene interaction data, and produces as output a dynamic regulatory map. The dynamic regulatory map highlights major branching events in the time series expression data and describes the transcription factors potentially responsible for them. The list of transcription factors can be found in Supplemental data 5.

To obtain better insight into the specific genetic processes that changed as a result of culturing cells in HS, we identified clusters of co-expressed genes by applying hierarchical clustering using z-scores (Supplemental Data 3) followed by PAM (partitioning around medoids) clustering (Fig. 3A). Six gene expression patterns emerged, and Gene Ontology (GO) terms associated with these six patterns where determined (Fig. 3, Supplemental Data 4). Changes in cell cycle and lipid metabolism gave the strongest signals related to the shift to HS. Most biological processes associated with the six clusters are consistent with our current and previous analyses^4^: cells become growth arrested (cluster 1, 4, 5) and lipid droplets, as well as VLDL secretion were increased (cluster 1, 6). We also observed a de-repression of apoptosis (cluster 2). Cancer cells like Huh7.5 often repress apoptosis, and the observed changes may reflect the loss of their proliferative character.

The Dynamic Regulatory Events Miner (DREM) allows one to model, analyze, and visualize transcriptional gene regulation dynamics^5,6^. The method of DREM takes as input time series of gene expression data and combines it with known transcription factor-gene interaction data. It produces as output a dynamic regulatory map. This map highlights major branching events in the time series expression data and describes the transcription factors potentially responsible for them.

The map produced by the DREM clusters the data in similar patterns as in the PAM clustering analysis, although we only found 5 clusters (Fig. 3B). This analysis shows two forks: the first occurs immediately, and appears to be directly related to the change of serum. In this analysis we used a very high threshold of significance (p < 10^−12^) for the processes and genes that had altered, but still found, in branch 1 and 2, over 150 transcription factors that are predicted to be linked to these changes (Supplemental data 5). Many of these transcription factors are liver, immune function, (lipid) metabolism and proliferation related.

A second fork occurs at day 8, from the branch 1, and transcription factors predicted to be driving these changes are similarly high in number (Supplemental data 5). A small number of those transcription factors also had elevated levels of transcription: AHR, MYC, EGR1 and DDIT3. AHR (aryl hydrocarbon receptor), is best known as the transcription factor regulating the cytochrome P450 genes, but has also been implicated in liver regeneration^7,8^, as a tumor suppressor^8^, in embryogenesis and numerous other processes, likely through its role in the regulation of eicosanoid metabolism, which are bioactive lipid mediators^9^. MYC (myelocytomatosis oncogene) is a multifunctional transcription factor that regulates cell cycle progression, apoptosis and cellular transformation. EGR1 (early growth response 1) functions as a transcriptional regulator. The products of target genes it activates are required for differentiation and mitogenesis^10,11^. EGR1 has been implicated in the regulation of cholesterol biosynthesis^12^. Studies suggest this is a cancer suppressor gene^10^. DDIT3 (DNA damage-inducible transcript 3), also known as C/EBP homologous protein (CHOP), is best known for its role in the unfolded protein response, and as a pro-apoptotic transcription factor during ER stress^13^. We have found no evidence of apoptosis in HS-cultured cells. Activation of DDIT3 in may therefore be involved in the response to massive transcriptional changes in HS cells: significant transcriptional changes in 1/3 of the genes would imply that many proteins need to be degraded, or newly synthesized. A role for DDIT3 has also been implied in the metabolic regulation through cAMP and the regulation of lipid metabolism^14^, and in differentiation of osteoblastic cells^15^.

These PAM clustering and DREM analyses both indicate a central role for cell cycle control, and linked to that, for the metabolic change. Thus, we further examined the metabolic changes occurring in HS-cultured cells that are predicted to accompany the transition from a proliferating to a differentiated and growth-arrested state of the cell.

### Metabolic reprogramming: reversal of the Warburg effect and metabolic diversification

Proliferating cells often display a ‘cancer metabolism’ profile, first described by Otto Warburg in 1924, which includes reduced levels of oxidative phosphorylation and mitochondrial activity, higher dependence on aerobic glycolysis and glutaminolysis for ATP production, and increased generation of biosynthetic intermediates that are essential for the production of macromolecules (phospholipids, nucleotides, proteins) to support cell proliferation^3,16–18^. The metabolic reprogramming that occurs during the Warburg effect is tightly regulated. Key regulators include pyruvate dehydrogenase kinase 1 (PDK1), the lactate dehydrogenase A/B ratio (LDHa/LDHb), and monocarboxylic acid transporter 4 (MCT4), as further explained in Supplemental Data 6.

To test our hypothesis that cancer metabolism is reversed in HS-cultured Huh7.5 cells we compared HS- to FBS-cultured cells using a combination of gene expression analyses, measurement of end-point metabolites and biological assays.

**Figure 4:**
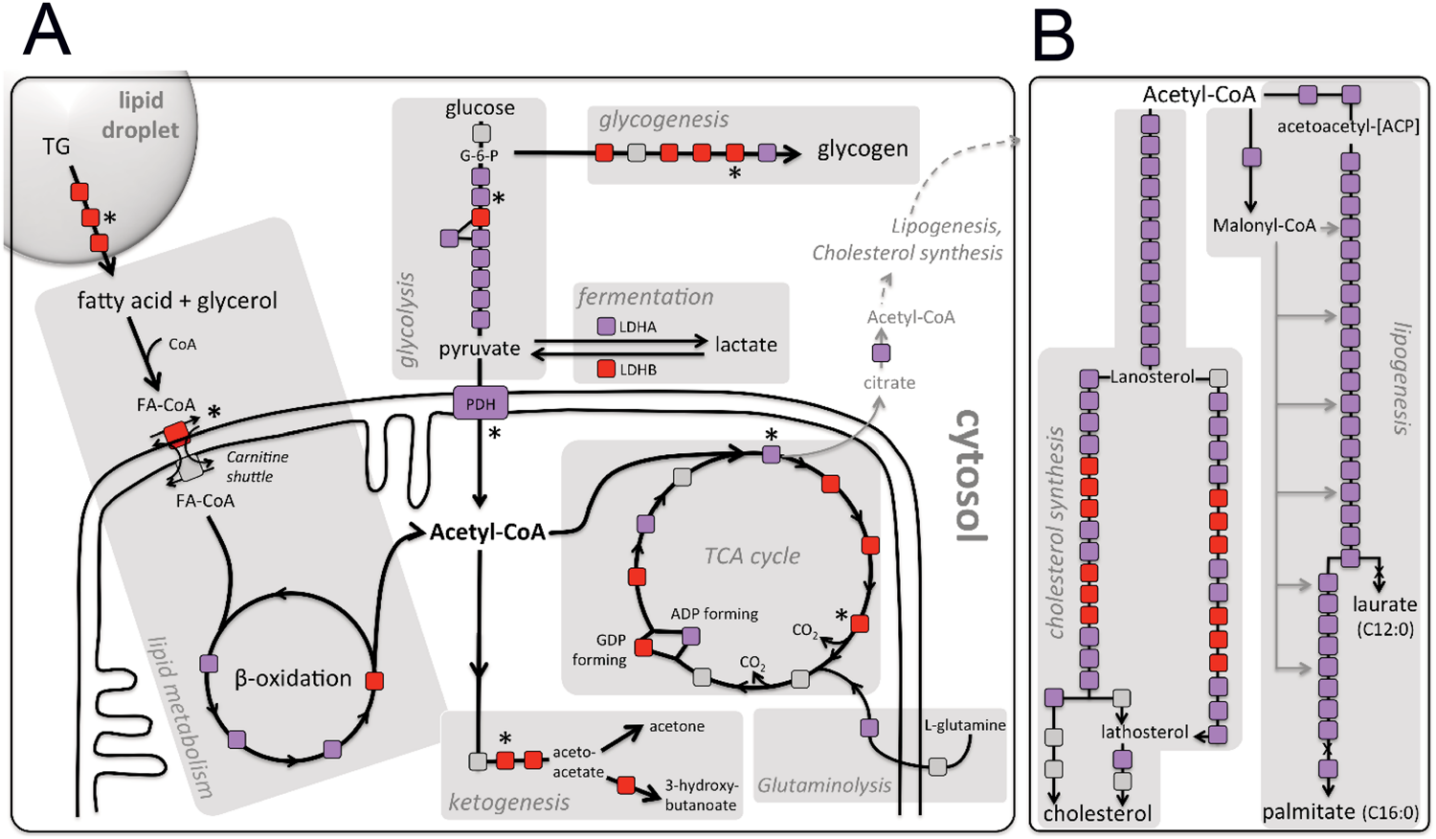
Metabolic mapping. Analysis of several metabolic pathways using HumanCyc: Encyclopedia of human genes and metabolism. Each enzyme in a biosynthetic pathway is depicted as a square. Changes in transcription levels are color-coded: red indicates an activated gene (P < 0.05), purple indicates a deactivated gene (P < 0.05) and grey indicates no significant change. Asterisks indicate the (predicted) rate limiting or rate regulating steps of the metabolic pathway. (**A**) Activity of the main ATP producing pathways in cells (**B**) cholesterol synthesis and lipogenesis.

**Figure 5:**
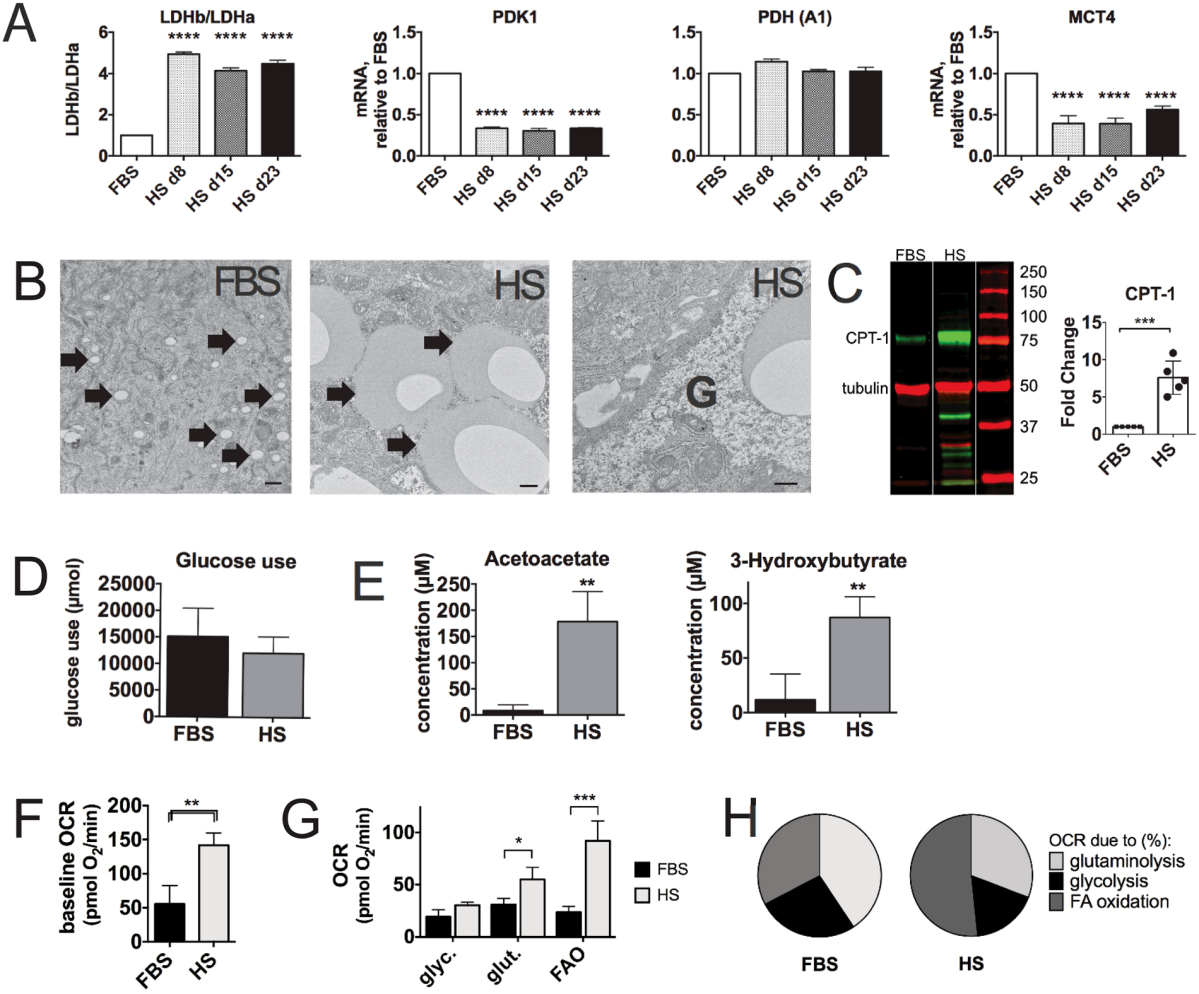
Metabolic reprogramming. (**A**) Reversal of the Warburg effect: Relative expression of genes involved in the regulation of the Warburg effect. Data were obtained from mRNA isolated a time series with 3 biological replicates each, and at least 2 measurements per biological replicate. Data are depicted as fold change over FBS. Depicted is mean with s.d., statistical significance was determined by ANOVA/Dunnett (one-sided, with multiple comparisons correction). (**B**) Lipid droplets (indicated by arrows) in FBS and HS-cultured cells, and presence of glycogen stores in HS-cultured cells (indicated by G). Shown are representative images from 3 separate experiments with 2 cell culture dishes each. (**C**) Licor® blot of CPT-1 (carnitine palmitoyl transferase-1) and tubulin. Shown is a representative image of 5 blots, the bands that are shown came from a single Licor® blot (for full length blot we refer to supplemental data 10). Quantitation of the fluorescence intensity of the 5 blots is represented in the right panel. Values are depicted as fold increase over FBS (n = 5, mean ± s.d.). Statistical significance was calculated using a t-test. (**D**) Quantitation of glucose use in FBS and HS cultured cells. (**E**) Quantitation of ketone bodies present in the cell culture supernatant of HS and FBS-cultured cells, by NMR. n = 4, depicted is mean ± s.d. Statistical significance was calculated using a t-test. (**F**,**G**) Live cell metabolism of FBS and HS cultured cells was determined using a Seahorse Biosciences XF Analyzer. (**F**) Total mitochondrial oxygen consumption rate is increased in HS cultured cells (n = 5 for FBS, n = 3 for HS, depicted is mean ± s.d.). (**G**) Capacity of FBS and HS cultured cells to use fatty acids, glucose and glutamine as energy sources. Depicted are the averages of actual oxygen consumption rates, with standard deviation. n = 5 for FBS, n = 3 for HS.). (**H**) Mitochondrial dependency on β-oxidation, glycolysis and glutaminolysis in FBS and HS cultured cells. The relative contribution of oxygen consumptions rates due to fatty acid oxidation, glucose metabolism and glutaminolysis, as fraction of the total OCR was calculated for each individual experiment. Depicted is the average over 5 experiments for FBS and 3 experiments for HS. P values ranges are depicted as asterisks: ^*^P < 0.05, ^**^P < 0.01, ^***^P < 0.001, ^****^P ≤ 0.0001, n.s: P > 0.05.

We investigated which metabolic pathways changed as Huh7.5 cells when they are cultured in HS instead of FBS, by visualizing microarray data on metabolic maps (www.HumanCyc.org). Figure 4A shows that fermentation and glutaminolysis are decreased in HS-cultured cells (day 23) relative to FBS cells, consistent with the reversal of cancer metabolism. In line with this, in HS-cultured cells the acidification of the cell culture media is much slower compared to their FBS counterparts, as indicated by medium color. Also, mRNAs of LDHa are decreased and LDHb is increased, shifting the reaction away from lactate production (Fig. 5A). mRNA of lactate transporter MCT4 is also decreased in HS-cultured cells, as is PDK1, which regulates pyruvate uptake by mitochondria (Fig. 5A, Supplemental Data 6). These data combined indicate a reversal of the Warburg effect.

Metabolic mapping additionally showed that (i) all but one glycolysis enzymes are down-regulated (Fig. 4A), including the rate-limiting step. The one enzyme that is up-regulated is also involved in gluconeogenesis, and the mRNAs of enzymes in the gluconeogenesis pathway were generally up-regulated (data not shown). (ii) Most glycogenesis enzymes, including the rate-limiting step are up-regulated (Fig. 4A), indicating that HS-cultured cells convert large amounts of glucose to glycogen, thus relying less on glucose for ATP production. This is supported by the presence of large glycogen deposits within the cell, as is shown in Fig. 5B (marked G). Glucose use was not altered in HS-cultured cell compared to FBS-cultured cells (Fig. 5D). (iii) Metabolic analysis supports an increase in β-oxidation rates: many enzymes involved in TG (Triacylglycerol) degradation and β-oxidation are increased, including the rate regulating step of β-oxidation, CPT-1 (carnitine-palmitoyl transferase 1; Fig. 4A). In HS-cultured cells lipid droplets size was increased (Fig. 5B, arrows, left and middle panel^4,19^). β-oxidation is in part regulated by the availability of lipid stores. Increased protein levels of CPT-1 (approximately 7-fold) were confirmed by western blot (Fig. 5C). (iv) Acetyl CoA produced by β-oxidation is partially converted to ketone bodies in the liver of healthy individuals, which play a critical role in normal energy homeostasis^20^. Formation of ketone bodies is therefore often used to estimate the rate of β-oxidation. Ketogenesis was increased in HS-cultured Huh7.5 cells (Fig. 4A), which was confirmed by NMR analysis of metabolic end-products in HS-cultured cells (Fig. 5E). Acetoacetate and 3-hydroxybuyrate, the two main ketone bodies produced during ketogenesis, were significantly increased in HS cultured cells (Fig. 5E), in line with increased β-oxidation rates in HS serum cultured cells. We further analyzed the metabolic pathways in live cells by measuring the capacity of mitochondria to use fatty acids, glutamine and glucose as substrates, by measuring the oxygen consumption rates (OCR) of mitochondria. In this analysis, the total mitochondrial OCR of cells in HS is approximately doubled, compared to cells in FBS (Fig. 5F). This is due to an increase in glutaminolysis in HS cells and a large increase in β-oxidation (Fig. 5G). When expressed as a fraction of the total mitochondrial OCR, this analysis shows that HS cells are predominantly dependent on β-oxidation for ATP production, at the cost of glycolysis, and to a lesser extent at the cost of glutaminolysis (Fig. 5H).

Finally, consistent with the reversal of a proliferative metabolic profile, mRNAs of pathways that produce ‘building blocks’ for cell growth and proliferation, specifically cholesterol synthesis and lipogenesis, are decreased in human serum (Fig. 4B). Citrate that is produced in the mitochondria can be exported to the cytosol, instead of utilized in the TCA cycle, where it is converted to Acetyl-CoA. Cytosolic Acetyl-CoA can then be incorporated into cholesterol and fatty acids, as depicted in Fig. 4B. mRNAs of most genes in these pathways are decreased in HS-cultured cells (day 23). Malonyl-CoA is also produced in the lipogenesis pathway and inhibits CPT-1, and thereby β-oxidation. Thus, its decreased production in HS-cultured cells is in line with the activation of β-oxidation in HS-cultured cells, and metabolic reprogramming in general.

Summarizing, our analyses show that the metabolism in HS-cultured cells shifts away from cancer metabolism (glutaminolysis and the Warburg effect) and glycolysis, in favor of glycogen storage. Lipid stores are increased, as are β-oxidation and ketogenesis.

### Xenobiotics biodegradation and metabolism

As cells move away from the state of proliferation and cancer metabolism and differentiation progresses, surplus nutrients may become available for storage, and to reinstate secretory processes that require large amounts of macromolecules, like VLDL secretion. Indeed, we showed previously that VLDL secretion is completely absent in FBS-cultured cells, but as cells become growth arrested and differentiate, VLDL secretion is gradually restored^4^. Here, KEGG (Kyoto Encyclopedia of Genes and Genomes) pathway enrichment analysis supports the re-establishment of lipid and carbohydrate metabolism as described in the previous sections, as well as re-establishment of secretory processes (Table 1, Supplemental Data 8). Enriched pathways included ‘steroid hormone synthesis’ (KEGG pathway hsa00140), ‘degradation of xenobiotics by cytochrome P450’ (KEGG pathway hsa00980) and ‘ascorbate and aldarate metabolism’ (KEGG pathway hsa00053). Steroid hormones derive from cholesterol and are secreted by various organs including the liver. Ascorbate and aldarate metabolism is central to many conversions of glucose, including nucleoside synthesis and pentose interconversions, which itself was significantly increased after 15 days in HS media. The most notable changes involved ‘Xenobiotics biodegradation and metabolism’. Three pathways in this cluster were significantly increased (Table 1) and several detoxification enzymes were present in the top-25 transcripts with increased expression. Notably, the enzyme with highest transcriptional increase was sulfotransferase 1E1 which catalyzes the sulfate conjugation of xenobiotics, facilitating removal (Supplemental Data 7).

**Table 1:** Significantly increased KEGG pathways (p < 0.05).

	KEGG ID	Pathway name	Fold change	p-value
HS D8	hsa00980	Metabolism of xenobiotics by cytochrome P450	3.65	0.0028
	hsa00140	Steroid hormone biosynthesis	3.90	0.0040
	hsa00053	Ascorbate and aldarate metabolism	4.27	0.0317
HS D15	hsa00140	Steroid hormone biosynthesis	8.18	6.16E-008
	hsa00980	Metabolism of xenobiotics by cytochrome P450	6.56	6.45E-007
	hsa00982	Drug metabolism - cytochrome P450	5.22	4.69E-005
	hsa00830	Retinol metabolism	5.30	0.0001
	hsa00983	Drug metabolism - other enzymes	4.89	0.0012
	hsa00053	Ascorbate and aldarate metabolism	6.52	0.0029
	hsa00040	Pentose and glucuronate interconversions	5.30	0.0063
	hsa00500	Starch and sucrose metabolism	3.92	0.0084
	hsa00860	Porphyrin and chlorophyll metabolism	3.94	0.0179
	hsa00514	Other types of O-glycan biosynthesis	3.68	0.0224
HS D23	hsa00980	Metabolism of xenobiotics by cytochrome P450	5.18	8.63E-007
	hsa00140	Steroid hormone biosynthesis	5.46	3.60E-006
	hsa00982	Drug metabolism - cytochrome P450	4.26	4.22E-005
	hsa00830	Retinol metabolism	4.42	6.90E-005
	hsa00053	Ascorbate and aldarate metabolism	5.44	0.0018
	hsa00983	Drug metabolism - other enzymes	3.81	0.0021
	hsa00040	Pentose and glucuronate interconversions	4.42	0.0048
	hsa00500	Starch and sucrose metabolism	3.14	0.0119
	hsa00860	Porphyrin and chlorophyll metabolism	3.29	0.0168
	hsa00514	Other types of O-glycan biosynthesis	3.08	0.0220

**Figure 6:**
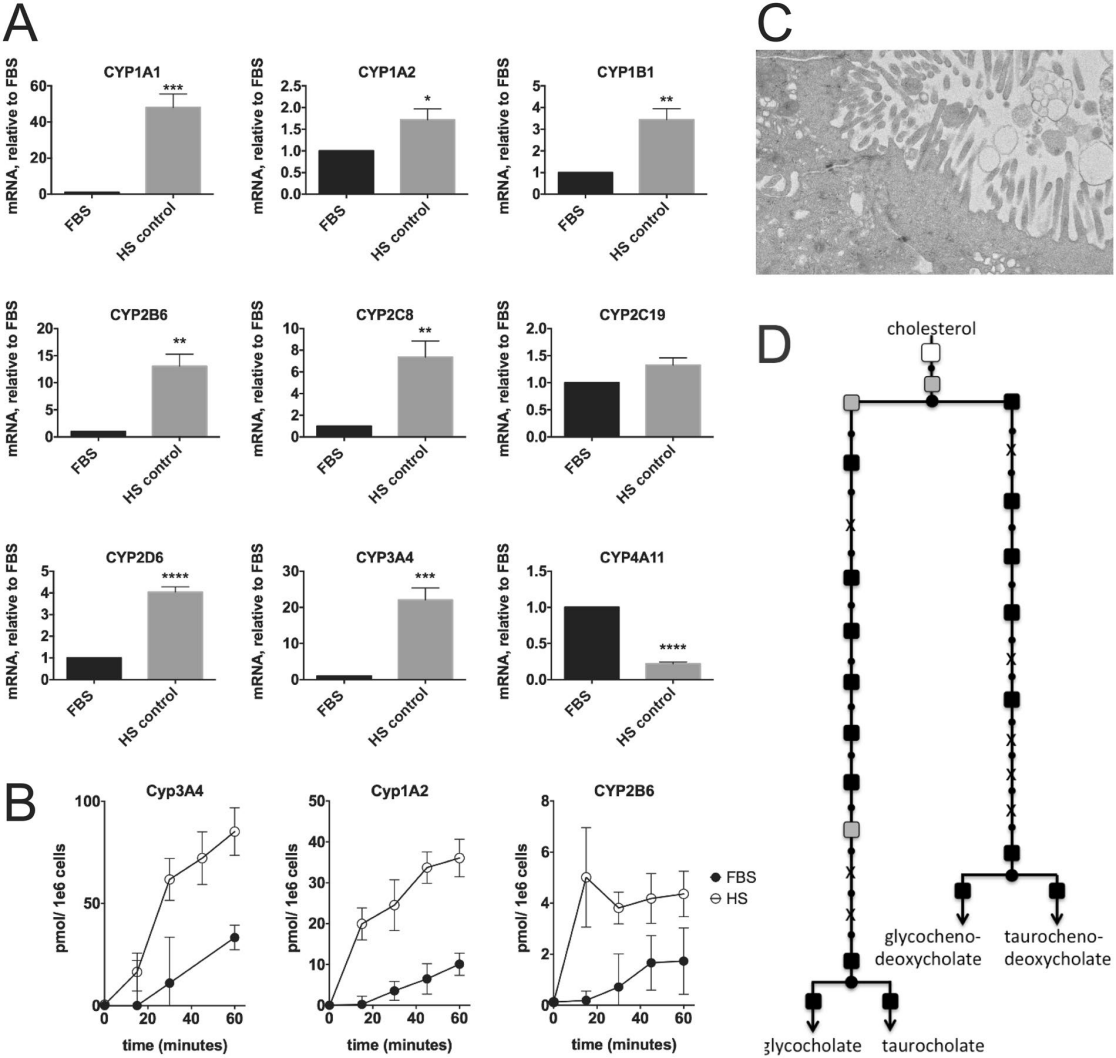
Cytochrome P450 metabolism and bile production. (**A**) mRNA of different cytochrome P450 (CYP) genes, as determined by quantitative PCR. Data are depicted as mean ± s.d. n = 4 (4 biological replicates with duplicate measurements each). Statistical significance was calculated using Student’s t-test. P values ranges are depicted as asterisks: ^*^P < 0.05, ^**^P < 0.01, ^***^P < 0.001, ^****^P ≤ 0.0001, n.s: P > 0.05 (**B**) Activity of selected CYPs, measured with a substrate cocktail assay. Shown is a representative time-series, of 3 independent experiments with duplicate cell culture dishes each. (**C**) Electron micrograph of a structure resembling a bile canalicular surface. Bar represents 500 nm. Shown is a representative figure from 2 experiments with 2 transwell dishes each. (**D**) Metabolic mapping of microarray data, of enzymes involved in bile acid synthesis. See the legend of Fig. 5A for further description of this analysis.

To confirm the increase in xenobiotics metabolic capacity, we focused on a panel of Cytochrome P450 (CypP450) enzymes, the key enzymes that degrade xenobiotics (Fig. 6A, Supplemental Data 9). All CypP450 enzymes had increased mRNA levels in HS (1.5–60 fold), with the exception of Cyp4A11. Cyp4A11 is involved in the detoxification of lipid products, when β-oxidation is absent or decreased. Thus, this finding is consistent with the observed increase in β-oxidation in HS-cultured cells. Transcriptional levels of other proteins involved in degradation and removal of xenobiotics were also determined, and findings were generally consistent with increased Cytochrome P450 activity, and degradation and removal of toxic compounds (Supplemental Data 9).

To functionally confirm the changes observed at a transcriptional level, we examined the rates of xenobiotics degradation in FBS- and HS-cultured cells (Fig. 6B). The intrinsic, non-induced rates of degradation by Cyp3A4 (testosterone), Cyp1A2 (methoxy-resorufin) and Cyp2B6 (bupropion) were increased approximately 4-, 6- and 6-fold respectively.

Many products resulting from CypP450 activity are removed from the liver in the bile, through the bile canalicular surface, which is only established in polarized cells. Bile synthesis is also dependent on the availability of excess cholesterol. Figure 6C shows the formation of structures that have a high resemblance to bile canaliculi in HS-cultured cells. Most mRNAs of enzymes involved in bile synthesis are increased (Fig. 6D), indicating higher levels of bile acid secretion. Similar observations were reported elsewhere in HepG2 cells which were cultured in human serum^21^.

## Discussion

When tissue and cell culture was initially developed in the early to mid 1950s, the lack of cell growth was a major problem, which was overcome by the addition of fetal animal sera or embryo extracts. The first human cell line for example, HeLa cells, was grown in chicken plasma clot, mixed with saline, human umbilical cord serum and bovine embryo extract^22^. Eventually, fetal bovine serum (FBS) became the serum of choice, because of its abundant availability and excellent growth promoting capacities. Undeniably, great progress has been made since cell cultures were first developed, however, it has also become increasingly clear that rapidly dividing cells are not representative of normal functional cells in an organ or organism, and can have an abnormal morphology of the cell or altered metabolism, and many lack organ-specific functionality.

In this study we used a human hepatocellular carcinoma cell line, Huh7.5, and showed that culturing these cells in their native adult serum, instead of FBS, results in growth arrest, drastic changes in morphology and intracellular organization, metabolic reprogramming and re-establishment of organ-specific functions. Significant changes in transcription in 32% of the genes (day 23) are indicative of extensive cellular reprogramming. The differentiation process takes approximately 3 weeks, which is in line with the time that is needed to differentiate stem cells^23,24^. We showed that metabolism shifts from a cancer-like profile, to a profile that is more representative of hepatic metabolism, that includes glycogenesis, higher β-oxidation rates, lower reliance on glycolysis rates, as well as restoration of processes like VLDL secretion^4^, degradation of xenobiotics by cytochrome P450s, and bile secretion (this study^21^). This debunks the notion that cell lines derived from cancers have lost their ability to undergo contact inhibition and some of their organ-specific functionality. We showed in Huh7.5 cells, that most of these lost functions, and a normal metabolic profile, are merely suppressed in the presence of a growth inducing serum, like FBS, and can be restored by culturing cells in their native adult serum. Other cell lines appear to undergo similar changes when cultured in their native adult serum, although this is not a universal effect. For example, HepG2, another hepatocellular carcinoma cell line, undergoes similar transitions as we described for Huh7.5 cells^21^; (unpublished observations), HeLa cells in our hands did not tolerate culturing in human serum, while A549 cells, a adenocarcinomic human alveolar basal epithelial cell line, did thrive in HS, stopped dividing and assumed an epithelial morphology.

The large number of transcription factors that are predicted to be involved in the differentiation (DREM analysis) supports a complex, multifaceted process, and it is highly unlikely that one or only a few factors in HS are responsible for transcriptional changes in 32% of the genes of the cells. We have, in this and in previous studies determined some of the (groups of) components in FBS or HS that likely play a role in the establishment of the different phenotypes: (i) the lack of growth factors in adult serum relative to fetal serum, (ii) the enrichment of certain differentiation inducting factors in HS, and (iii) the different lipid composition of human relative to bovine sera.

Most cell cultures need fetal sera to proliferate, as they are enriched in proliferation promoting factors. In line with this, cell proliferation slows down immediately after Huh7.5 cells are transferred to HS, and completely stops after 7–10 days. We do not believe that the difference in serum concentration (10% FBS vs 2% HS) is responsible for the growth arrest, as cells also stop proliferating in 10% HS. As human serum is costly, and there is no obvious benefit of using higher concentrations of serum, we use 2% human serum. 2% FBS has been used to starve cells and induces cell cycle arrest in various cell lines, but it does not induce cell differentiation and the restoration of processes like VLDL secretion in Huh7.5 cells. Instead it causes cell death when used for prolonged culturing (more than a week). Cells cultured in HS can be kept for at least 100 days, without subculturing^4^, and build up large glycogen and lipid stores, indicating that they are thriving.

The DREM analysis and PAM clustering both indicate a central role for cell cycle control in the early stages of HS induced differentiation. Growth factors also regulate oncogenes, which in turn influence metabolism, e.g. oncogenes c-Myc and p53 directly regulate mitochondrial respiration^25,26^ and are amongst the most common alterations in human cancers, including in Huh7.5 cells^27,28^. c-Myc increases LDHa, whereas the tumor suppressor p53 stimulates oxidative phosphorylation and controls the rate limiting step of glycolysis^25,26^. Thus, the lack of oncogene stimulation, through the reduced levels of growth factors in adult serum (like HS) might play a direct role in the observed metabolic reprogramming. Similarly, inhibition of the Ras-MEK-ERK pathway, for example by using MEK inhibitors, is linked to decreased glycolytic flux and decreased lactate production^29,30^, which occurs through interaction with an intermediate of glycolysis, fructose-1,6-bisphosphate. MEK-ERK inhibition was also used to improve VLDL assembly and secretion in HepG2 cells^31^, possibly through the induction of metabolic changes (e.g. the reversal of the Warburg effect) which occur upon MEK/ERK inhibition, or alternatively through a direct role of ERK in VLDL assembly^31^.

Factors that are specific to or enriched in human sera may also play a role. For example, Huh7.5 cells that are cultured in *adult* bovine serum (ABS), also undergo growth arrest and up-regulate tight junction and cell-cell contact components^4^, but these cells otherwise lack hepatocyte functionality like albumin and VLDL secretion. Conversely, human umbilical cord blood serum (CBS), the closest human equivalent of FBS, induces high cell proliferation rates, but also increases Huh7.5 functionality as shown by increased levels of albumin secretion. Like HS cultured cells, CBS cultured cells can support the production of very high levels of hepatitis C virus, without inducing cell lysis^32^. VLDL secretion is not restored in CBS-cultured cells^32^, and possibly VLDL secretion co-depends on the presence of sufficiently large TG stores (lipid droplets), and thus may co-depend on a non-proliferative metabolic profile/growth arrest.

Some differences in growth factor composition in bovine sera and human sera have been determined. Growth factors that are remarkably higher in human serum (CBS and HS), compared to FBS, are IGF-1 (insulin like growth factor 1) and several IGFBPs (IGF binding proteins)^33^. IGF-1 is increased approximately 20 and 40-fold in HS and hUCBS respectively, relative to FBS. IGF-1 and IGFBPs both play a role in hepatocyte differentiation, with IGFBPs modulating and prolonging the activity of IGF-1. Consistent with their role in differentiation, IGF-1 and IGFBPs impede the aggressive growth of certain liver cancers^34–36^. IGFBP-3 is one of the factors listed in the top-25 genes with the greatest increase in transcription in our microarray analysis (Supplemental Data 7).

The composition of the lipids of HS is also markedly different from FBS. Lipoproteins in bovine serum mainly contain saturated fatty acids, whereas lipoproteins in human serum are enriched in lipids containing unsaturated fatty acids, particularly in oleic acid (18:1), arachidonic acid (20:4) and the essential fatty acid linoleic acid (18:2)^37^. Oleic acid is often added to cells to stimulate lipoprotein secretion^38^, which cannot be achieved by addition of saturated fatty acids, and the presence of this fatty acid in human serum may thus further facilitate the restoration of VLDL secretion. Arachidonic acid is the precursor of eicosanoids, signaling molecules made by the enzymatic or non-enzymatic oxidation of arachidonic acid or other polyunsaturated fatty acids (PUFAs). Eicosanoids include prostaglandins, leukotrienes and lipoxins, bioactive lipid mediators that are involved in the complex control of virtually all life functions^9^. Arachidonic acid is also a natural ligand of the aryl hydrocarbon receptor (AHR)^39^. DREM analysis of our data indicated that AHR is upregulated in HS cultured cells, and AHR appears to play a role in the multiple aspects of the differentiation process. The aryl hydrocarbon receptor is a ligand-activated transcription factor that plays numerous important endogenous roles: during conception and embryonic and fetal development; in the immune systems; and, as mentioned, in the activation of Cytochrome P450 in hepatocytes^9^. Increased cytochrome P450 activity in its turn, as determined by the KEGG analysis, further facilitates eicosanoid production, which are known to undergo Cytochrome P450 mediated modification^9^.

Summarizing, we have shown that simply (and only) changing the serum source can drastically change the functioning of cells. Based on our current knowledge we believe that at the base of this differentiation is the lack of growth factors in adult human serum, which induces growth arrest, the formation of tight junctions and the reversal of the Warburg effect. Other serum factors, some of which we have described, are predicted to be involved. They likely further facilitate morphological changes and metabolic diversification over time, and eventually the restoration of hepatocyte specific functions.

Given the critical importance of morphology and metabolism in a myriad of processes, HS-based cell culturing may increase the usefulness of human cancer cell lines by establishing affordable, scalable and physiologically relevant cell culture models. These cells enable the study of normal physiology and the transition to the diseased state, for example the formation of fatty liver disease and progression of cancers. Mostly, the profound changes we observed, simply as a result of changing serum in cell cultures where surprising to the authors, and the detailed study we presented here, combined with previous studies, provides important insight in the effects and relevance of serum choice in any study.

## Methods

### Cell cultures

Huh7.5 cells and were a gift of Dr. C. Rice (Rockefeller University, New York, USA). Normally, for proliferation, cells were maintained in DMEM (Sigma Aldrich D5796; high glucose, with L-glutamine and sodium bicarbonate, without sodium pyruvate) supplemented with 10% FBS and penicillin/streptomycin, as described previously^4^. Cells were usually replated at a density of 33% and never replated at a density of less than 25%. The cell line used in this study has a low passage number, and after approximately 30 passages, cells were discarded, and a new vial was thawed.

### Differentiation of cells in human serum

Huh7.5 differentiation using HS has been described previously^4^. For our studies we use pooled human AB serum (Valley Biomedical (Winchester, Virginia) Sigma-Aldrich, or any other reliable provider). Pooled human serum is prepared from the serum from 80–130 healthy donors, which removes the variability we observe with individual serum samples. Since the use of human serum results in growth arrest^4^, cell cultures were normally maintained in FBS-containing media as described above^4^. At the time of transfer to human serum, cells were trypsinized, trypsin was inactivated with DMEM/10% FBS/penicillin/streptomycin and cells were centrifuged at 300 g. Cell pellets were then resuspended in DMEM/2% HS/penicillin/streptomycin, and plated at a density of 30–50%^4^ on Eppendorf cell culture plates. At confluency cells were trypsinized once more, and plated at a density of 50%. They then form confluent layers of undividing cells^4^.

The differentiation process takes approximately 21 days. Cells can be trypsinized/sub-cultured for approximately 7–10 days, but continued sub-culturing beyond that leads to cell death, as the cells do not attach to cell culture plastic any more^4^.

### Microarray analysis

Cells were cultured (3 biological replicates/flasks) either in FBS, or in human serum for 8, 15 or 23 days. These days were chosen because HS-cultured cells undergo growth arrest around day 7–10 post transfer, the first morphological signs of differentiation appear around day 14, and the differentiation process appears complete after 21 days in human serum containing media. Cell lysates were then prepared for microarray analysis of Affymetrix GeneChip® PrimeView™ Human Gene expression Array cartridges according to the instructions of the manufacturer. Microarray data were deposited in the GEO repository with Accession Number GSE87684 (GEO).

All expression data analyses were performed using the R statistical program (R Development Core Team; R: A Language and Environment for Statistical Computing (R Foundation for Statistical Computing)^40^)^40^). Probe intensities were normalized with the “affy” R package from the Bioconductor project^41^, using the “Robust Multiarray Averaging” (RMA) algorithm^42^. Differential expression analysis was performed using the “limma” R package^43^, which fits a linear model to the expression data. Principal Components Analysis was performed using the R “prcomp” function. For the clustering analysis gene expression levels were converted to z-scores, standardized across the samples, to eliminate the differences in absolute gene expression levels, and focus on the pattern of changes. An initial hierarchical clustering was performed using Euclidean distances and average linkage in order to get an idea of how many clusters were present in the data. Based on this the expression data were clustered into 6 clusters using the Partitioning Around Medoids (PAM) algorithm, which requires a predetermined number of clusters to be specified. Gene Ontology term enrichment calculations were performed using the “goseq” R package^44^.

### Metabolic modeling

For our metabolic modeling purposes we use a recently constructed stoichiometric metabolic network for human, called Recon2^45^. The boundaries of exchange reactions were left at default values and dead-end reactions (i.e. the reactions whose product metabolites are not used for any other reaction or for growth) were removed. We integrate the measured gene expression values with this model using a program called Metabolic Adjustment by Differential Expression (MADE)^46^. MADE uses the p-values and fold changes obtained in a differential gene expression analysis to identify enzymes with significantly different expression between conditions, and looks for the best fit with the metabolic network. The program predicts for each gene if it is activated, deactivated or stable between conditions and time points, such that each prediction is consistent with the network structure. This can be visualized as activation and inactivation of the corresponding reactions using the online tool HumanCyc^47^.

### Transmission Electron microscopy (TEM)

For conventional transmission electron microscopic study, cells were either cultured in FBS and grown to confluence, or cultured in HS-containing media, grown to confluence and allowed to differentiate. Some FBS and HS-cultured cells were grown on a membrane of BD Falcon cell culture inserts (cat. #353180, BD Biosciences, Canada), in order to obtain perpendicular sections of cells on the membrane surface. Cells were then prepared for electron microscopic analysis as described before^48^ with minor modifications: 2x conventional TEM fixative (mixture of glutaraldehyde (4%), paraformaldehyde (2%), sucrose (0.2 M) and CaCl_2_ (4 mM), in 0.16 M sodium cacodylate buffer, pH 7.4) was added to the cell media to make optimally diluted fixative. Pre-fixation was performed at 37 °C for 1 hour. Following pre-fixation, cells were washed with 0.05 M sodium cacodylate buffer (cat. #11654, Electron Microscopy Sciences, USA) to remove residual aldehyde from cells. For lipid fixation, cells were post-fixed with 1% ice-cold osmium tetroxide (OsO_4,_ cat. #19140, Electron Microscopy Sciences, USA) in 0.05 M sodium cacodylate buffer. Next, cells were washed with 0.05 M sodium cacodylate buffer. To enhance contrast of cell membrane and subcellular membrane (en bloc stain), cells were treated with 1% uranyl acetate (cat. #22400-4, Electron Microscopy Sciences, USA) in 0.1 M sodium acetate buffer (pH 5.2) for 15 min. Cells were washed with 0.1 M sodium acetate buffer followed by Milli-Q filtered water and then dehydrated with ascending ethanol series (30, 50, 70, 80, 90, 95 and 100%). Cells on membranes were gradually infiltrated with Spurr’s resin (cat. #14300, Electron Microscopy Sciences, USA). Several pieces from the membranes of the cell culture inserts were embedded into BEEM flat embedding molds (cat. #7004-01, Electron Microscopy Sciences, USA), so that they could be sectioned perpendicular to the membrane surface and polymerized at 65 **°**C for 24 h. Ultra-thin sections with a thickness of 60 nm from polymerized resin blocks were sectioned using a Leica EM UC7 ultramicrotome (Leica Microsystems Inc. Canada), transferred on bare Cu grids, and post-stained with uranyl acetate (2%) and Reinolds’ lead citrate for 10 minutes each. Sections were observed under a Hitachi H-7650 transmission electron microscope (Hitachi-High Technologies Canada) at 80 kV and imaged under a high definition electron multiplying charge coupled device (EMCCD) camera (XR111, Advanced Microscopy Techniques, USA).

### Immunofluorescence staining

For confocal imaging of claudin-1, tubulin and vimentin, cells were grown in FBS or in HS-containing media coverslips coated with poly-L-lysine. Cells were washed with PBS, and fixed in 3.65% formaldehyde in PBS for 8 minutes at room temperature. After fixation cells were washed 3x with PBS, incubated with 0.2% Triton-X-100 for 2 minutes at room temperature, and washed again 3x with PBS, followed by a 2 hour blocking step in PBS containing 0.1% Tween-20 and 2% milk powder. Primary antibodies (anti-α-tubulin: Sigma-Aldrich, T6074; rabbit-anti-claudin-1: Invitrogen 71–7800; mouse anti-vimentin (v9): Abcam Ab8069), diluted in 0.1% Tween-20 PBS containing 2% milk, were added to the cells and incubated overnight at 4 °C. Cells were washed the next day, 3×, in PBS containing 0.1% Tween-20 followed by incubation with Alexa Fluor conjugated secondary antibodies AlexaFluor488 donkey anti-mouse IgG (Life Technologies, A21202), AlexaFluor594 donkey anti-mouse IgG (Life Technologies, A21203), AlexaFluor594 donkey anti-rabbit IgG (Life Technologies, A11012) for 45 minutes at room temperature. After washing the cells 3x in PBS 0.1% Tween-20, cells were incubated in a Hoechst stain, 1:3000 for 5 minutes and coverslips were mounted to slides using Fluoromount-G (SouthernBiotech). Confocal images were obtained with a LSM 710 Axio Observer microscope (Carl Zeiss Inc.) using a 63×/1.40 NA Oil DIC Plan-Apochromat objective. Images were acquired as a z-stack series (with a distance of 0.24 μm between slices) and are represented as a z-projection.

### Dextran diffusion studies

Cells were grown on collagen coated transwell dishes and in the case of HS-cultured cells allowed to differentiate, or in the case of FBS-cultured cells grown to confluency. Cells were then place in phenol red free media, and 1 µg/ml 70 kDa dextran conjugated to Oregon green was added to the top compartment. Samples were taken every 30 minutes, for a total of 2 hours, from the bottom compartment to determine dextran diffusion rates. Oregon green Fluorescence was measured on an Enspire 2300 Multilabel reader (Perkin Elmer) and compared to subconfluent FBS-cultured cells to determine maximal diffusion rates. Confluency of FBS-cultured cells was ensured by visualizing cells on filters under a conventional phase-contrast microscope, as well as measuring FBS diffusion rates on 3 consecutive days. The lowest diffusion rate of those 3 was used, if differences existed.

### NMR analysis of reporter metabolites

Target profiling and analysis of reporter metabolites by NMR^49^ was performed by Chenomx Inc., Edmonton, Canada, according to the methods provided by the company and as previously described^50^. In short, FBS-cultured cells and confluent, differentiated HS-cultured cells were washed with DMEM and placed in DMEM (without serum) overnight. Supernatants of HS or FBS-cultured cells were collected, first filtered through 22 µm filters to remove large debris. Internal standard solution (IS-1) was added to each sample, and the resulting mixture was vortexed for 30 seconds. Samples were then filtered through Nanosep 3 K Omega microcentrifuge tubes to remove all proteins and other large complexes, and transferred to an NMR tube for data acquisition.

NMR spectra were acquired on a Varian two-channel VNMRS 600 MHz NMR spectrometer equipped with an HX 5 mm probe. The pulse sequence used was a 1D-tnnoesy with a 990 ms presaturation on water and a 4 s acquisition time. Spectra were collected with 32 transients and 4 steady-state scans at 298 K. Spectra were then processed using the Processor module in Chenomx NMR Suite 8.0. Compounds were identified and quantified using the Profiler module in Chenomx NMR Suite 8.0 with the Chenomx Compound Library version 9, containing 332 compounds.

### Seahorse Bioscience metabolic flux analysis

The cellular capacity of glycolysis versus β-oxidation was measured using a Seahorse XF analyzer, using the Seahorse XF Mito Fuel Flex Test Kit, according to the instructions provided by the supplier. We analyzed our data using the report generator provided by Seahorse Bioscience available on their website, with normalization to cell number. In short, after establishment of the baseline oxygen consumption rate (baseline OCR), the inhibitors for long chain fatty acid oxidation (etomoxir), glycolysis (UK5099) or glutaminolysis (BPTES) were added to separate wells. This allows the measurement of the target OCR, to establish the oxygen consumption attributed to each of these three metabolites. Subsequently the two other inhibitors are added to each well to establish the residual OCR, representing non-mitochondrial oxygen use and mitochondrial oxygen use that is not inhibited by the 3 inhibitors, such short chain fatty acid oxidation. The total mitochondrial OCR is determined by subtracting the residual OCR from baseline OCR.

### RNA extraction cDNA synthesis

RNA isolation and cDNA was prepared as described previously^51^. RNA was isolated from cells using TRIzol reagent^52^ (Invitrogen) according to the protocol provided by the supplier. 1.5 μg of total RNA was used for each sample. First-strand cDNA synthesis was performed by using the High-Capacity cDNA reverse transcription kit (Applied Biosystems) according to the manufacturer’s instructions and as previously described^51^.

cDNA was then quantitated by Reverse transcriptase/real time-PCR^53,54^, using a Bio-rad CFX-96 Real-Time PCR System (Bio-rad), using Bio-rad chemistry. The primers used in the current study were chosen from previously published studies^55^. No-template controls were also included on the same plate. At the end of each cycle, dissociation curves were determined, to confirm the specificity of the primers and the purity of the final PCR product. The real time-PCR data were analyzed using the relative gene expression method^50^ and presented as the fold change in gene expression normalized to the endogenous reference gene (β-actin) and relative to the untreated control of the same time point^51,56^.

### Measuring different P450s activities using cocktail substrates

The reaction was started by incubating live intact cells with 200 µL William’s E Medium containing a mixture of substrates including, 100 µM 7-methoxyresorufin (CYP1A2 substrate), 500 µM bupropion (CYP2B6 substrate), 20 µM paclitaxel (CYP2C8 substrate), 250 µM S-mephenytoin (CYP2C19 substrate), 15 µM dextromethorphan (CYP2D6 substrate), 200 µM testosterone (CYP3A substrate) in addition to 20 unites of sulfatase and 5000 units of β-glucuronidase. To stop the reaction 200 µL methanol was added, containing 100 µM acetaminophen as internal standard for LC/MS analysis.

The formed metabolites were then analyzed on a liquid chromatography–tandem mass spectrometry system, similar to a previously published method^57^. Selected reaction monitoring in the positive-ion electrospray ionization mode was performed for acetaminophen as an internal standard, resorufin (a metabolite of 7-methoxyresorufin produced by CYP1A2 activity), hydroxybupropion (a metabolite of bupropion produced by CYP2B6 activity), 6α-hydroxypaclitaxel (a metabolite of paclitaxel produced by CYP2C8 activity), 4′-hydroxymephenytoin (a metabolite of S-mephenytoin produced by CYP2C19 activity), dextrorphan (a metabolite of dextromethorphan produced by CYP2D6 activity), and 6β-hydroxytestosterone (a metabolite of testosterone produced by CYP3A activity).

### Data availability, code availability

Microarray data were deposited in the GEO repository with Accession Number GSE87684 (GEO), other source data are available in the supplemental data. Code is available from the authors upon request.

### Statistical analysis

All experiments consisted of a minimum of 3 independent experiments, as outlined in the figure legends). The Prism Statistics package was used for statistical analysis, as described previously^4^. Statistical significance was calculated using ‘Student’s t-test (unpaired, two-tailed) or one-sided ANOVA/Dunnett (with adjustments for multiple comparisons) as indicated in the legends. Values are depicted as means, with s.d. or s.e.m. P-values < 0.05 were considered significant. P values ranges are depicted as asterisks: ^*^P < 0.05, ^**^P < 0.01, ^***^P < 0.001, ^****^P ≤ 0.0001, n.s: P > 0.05.

## Electronic supplementary material

supplemental figures

Supplementary data 4

Supplementary data 8
