# Supplementary material for: Establishing normal metabolism and differentiation in hepatocellular carcinoma cells by culturing in adult human serum
Source: Sci Rep. 2018 Aug 3;8:11685. doi: 10.1038/s41598-018-29763-2 (PMC6076254; doi:10.1038/s41598-018-29763-2)
Supplement: Supplementary file 1 — supplemental figures [file 41598_2018_29763_MOESM1_ESM.pdf]

Supplemental data

Establishing normal metabolism and differentiation in hepatocellular carcinoma cells by culturing in adult human serum.

Rineke Steenbergen, Martin Oti, Rob ter Horst, Wilson Tat, Chris Neufeldt, Alexandr Belovodskiy, Tiing Tiing Chua, Woo Jung Cho, Michael Joyce, Bas E. Dutilh, D. Lorne Tyrrell

Supplemental Data 1

Other morphological changes in HS cultured cells

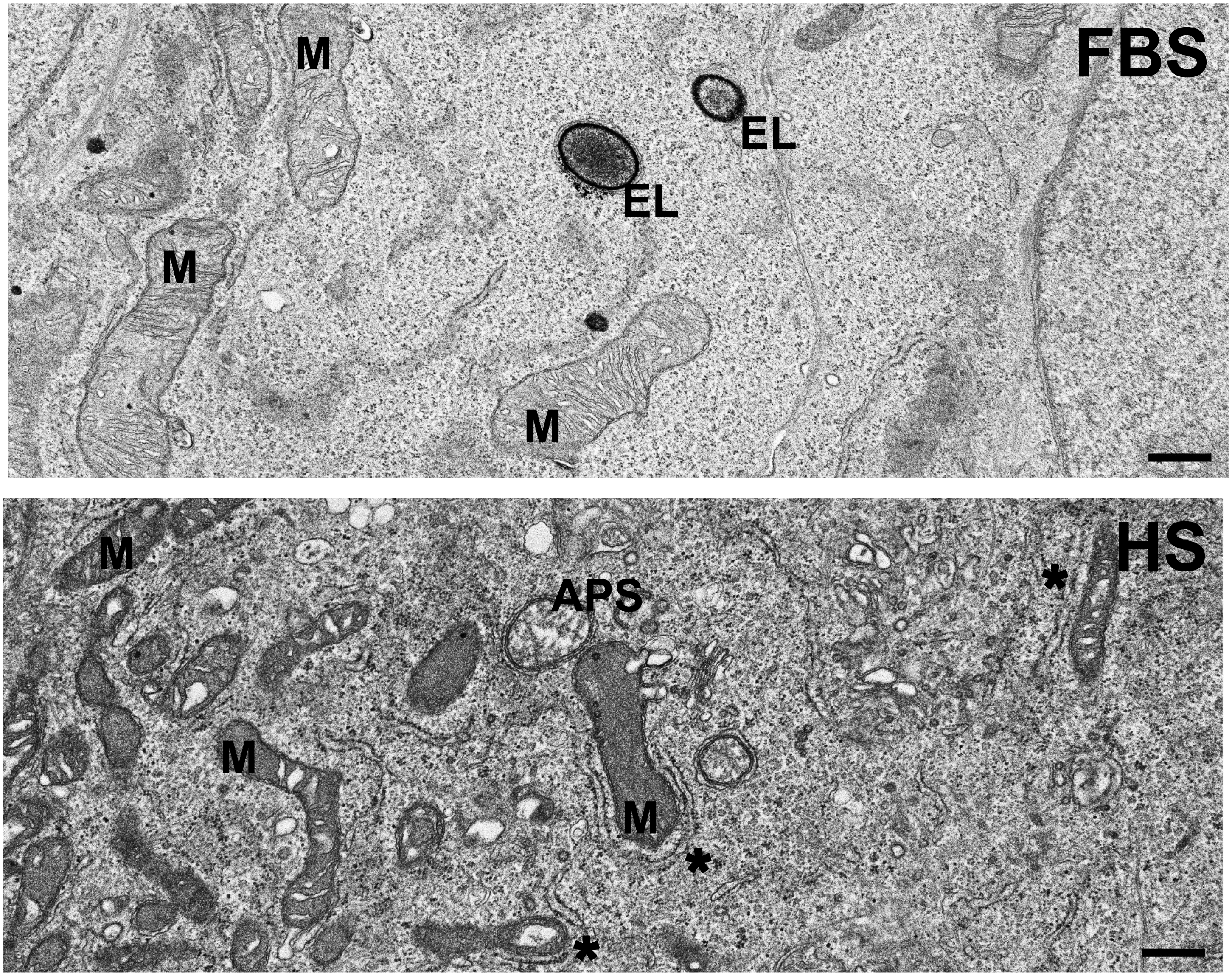

Additional morphological changes that were observed in the electron microscopic analysis include:

- The cytoplasm of HS cultured cells appears much more 'crowded' than the cytoplasms of FBS cultured cells.

- Mitochondrial (M) morphology has drastically changed, as outlined in figure 4 of the main document.

- In general. organelles appeared more structured and organized, for example, the endoplasmic reticulum (asterisks) shows a higher degree of organization, particularly around the mitochondria.

- An increase in vesicle transport and changes in autophagy and the lysosomal pathways were also observed on the electron micrographs. Whereas in FBS cells early lysosomes (EL) are abundant, other components of the endosomal/lysosomal pathway were harder to find, and no evidence was found of autophagy. In HS cultured cells late endosomes, multivesicular bodies and autophagosomes (APS) where abundant, potentially indicating a better functioning or more balanced endosomal/ lysosomal/ autophagosomal pathway is operational in HS cultured cells.

Supplemental Data 2

**Comparison**	**Number of differentially expressed probes (multiple testing corrected, $p<0.05$)**	**%**
FBS - HSd8	16304	33
FBS - HSd15	11420	23
FBS - HSd23	16252	33

Number of differentially expressed probes for the different pairwise comparisons between days. The total number of probes on the Primeview Array was 49395.

Supplemental data 3, hierarchical clustering analysis

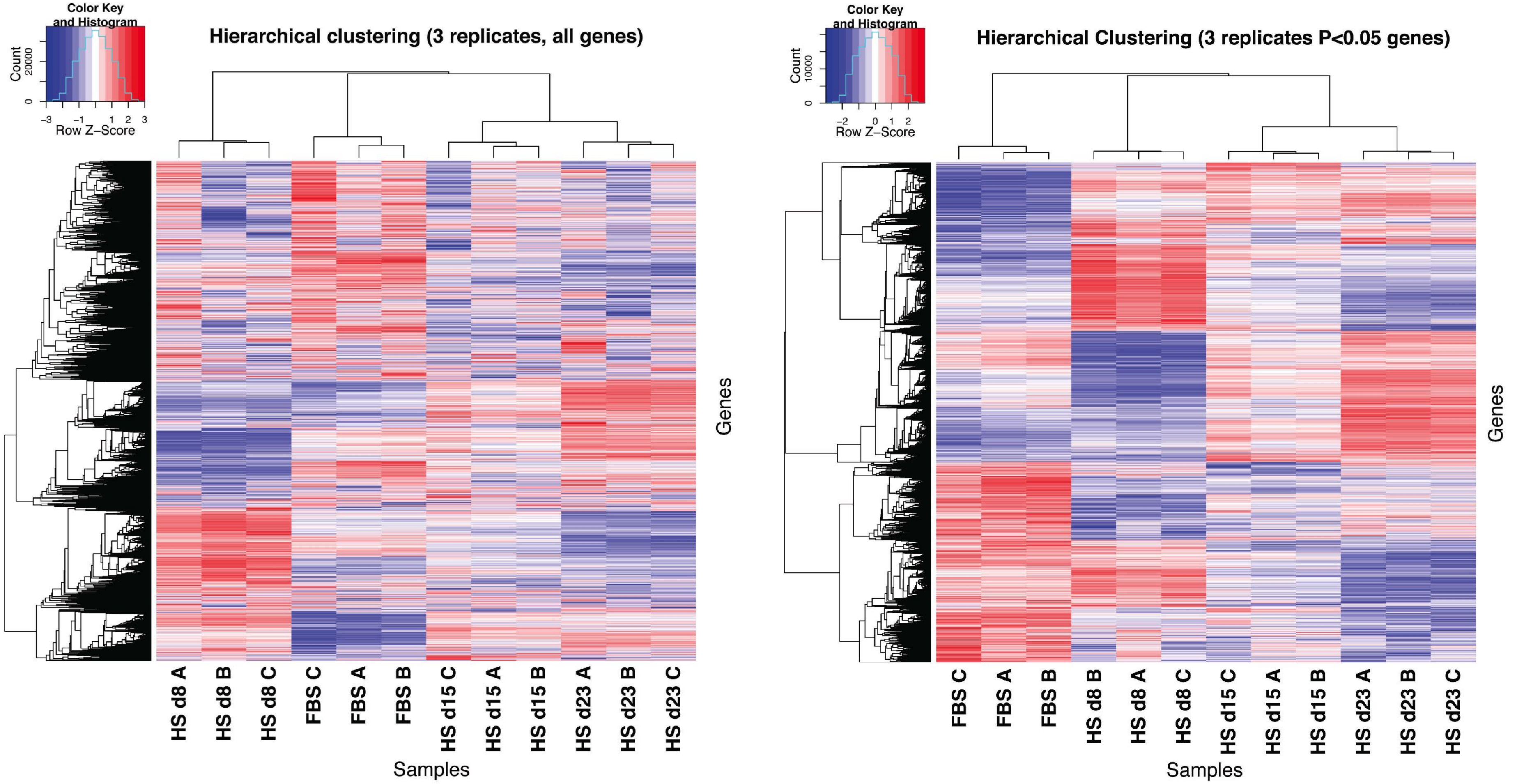

Clustering is a machine learning algorithm that groups data that have a high degree of similarity together in a cluster. In this analysis, data were clustered, initially, by using hierarchical clustering as shown above, based on gene expression pattern similarity of the entire dataset (y-axis) as well as on patterns of expression over time (x-axis). The z-scores of the gene expression levels were used in order to focus on the expression variation pattern across the samples rather than on their absolute expression levels, which can vary strongly from gene to gene.

Hierarchical clustering of the entire data set is shown on the left and of transcripts that showed a significant change ($p < 0.05$) is shown on the right In both analyses the replicates clustered together (x-axis), as was also shown by the PCA analysis (figure 1), and 6 patterns emerged (y-axis) when the data with significant changes were considered (right panel). To further analyze the genes or processes that were associated with these 6 clusters, a variant of k-means clustering was used, PAM clustering (partitioning around medoids), to generate more clearly delineated clusters (see figure 3 in the main document).

Supplemental Data 5: DREM analysis

The Dynamic Regulatory Events Miner (DREM) allows one to model, analyze, and visualize transcriptional gene regulation dynamics. The method of DREM takes as input time series gene expression data and known transcription factor-gene interaction data, and produces as output a dynamic regulatory map. The dynamic regulatory map highlights major branching events in the time series expression data and described the transcription factors (TFs) potentially responsible for them.

A: TFs that were significantly upregulated and predicted to be associated with upregulated pathways (p<10e-12).

B: TFs predicted to be associated with upregulated pathways (p<10e-12) (in order of significance for the associated processes).

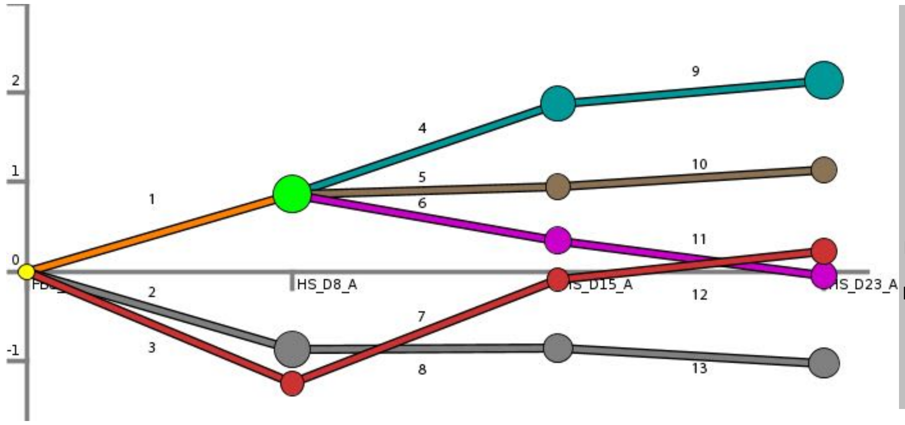

predicted associated PAM cluster

I translation
lipid metabolism

II cell membrane processes, apoptosis

III macromolecule metabolism (RNA, protein)

IV macromolecule metabolism (protein)
G1/S checkpoint

V cell cycle
cholesterol metabolism

	FBS to Day 8			DAY8 TO DAY 15					DAY15 TO DAY 23				
	1	2	3	4	5	6	7	8	9	10	11	12	13
A					AHR<br>MYC<br>EGR1<br>DDIT3	AHR	AHR	MYC<br>AHR		AHR<br>MYC<br>DDIT3	AHR	AHR	MYC<br>AHR

	1	2	3	4	5	6	7	8	9	10	11	12	13
B	RXRA	POU2F1	CUX1	MEF2A	RXRA	POU2F1	CUX1	POU2F1	MEF2A	RXRA	POU2F1	CUX1	POU2F1
	POU2F1	HNF1A	POU2F1	POU2F1	MEF2A	CUX1	POU2F1	HNF1A	POU2F1	MEF2A	CUX1	POU2F1	HNF1A
	CUX1	CUX1	MEF2A	RXRA	POU2F1	GABPA	MEF2A	CUX1	RXRA	POU2F1	GABPA	MEF2A	CUX1
	MEF2A	POU3F2	TCF3	IRF1	PPARG	GATA1	TCF3	POU3F2	IRF1	PPARG	GATA1	TCF3	POU3F2
	HNF1A	RXRA	STAT1	SRF	STAT1	HNF1A	STAT1	RXRA	SRF	STAT1	HNF1A	STAT1	RXRA
	STAT1	NR3C1	STAT4	HNF1A	CUX1	TCF3	STAT4	NR3C1	HNF1A	CUX1	TCF3	STAT4	NR3C1
	TCF3	STAT1	RXRA	CUX1	HNF1A	RXRA	RXRA	STAT1	CUX1	HNF1A	RXRA	RXRA	STAT1
	SRF	MEF2A	CEBPA	NR3C1	TCF3	STAT1	CEBPA	MEF2A	NR3C1	TCF3	STAT1	CEBPA	MEF2A
	PPARG	NFYA	SRF	TCF3	POU3F2	TBP	SRF	NFYA	TCF3	POU3F2	TBP	SRF	NFYA
	IRF1	PGR	PDX1	PPARG	CREB1	ELK4	PDX1	PGR	PPARG	CREB1	ELK4	PDX1	PGR
	POU3F2	CEBPA	FOXL1	NRIH4	STAT5A	TEAD1	FOXL1	CEBPA	NRIH4	STAT5A	TEAD1	FOXL1	CEBPA
	STAT5A	NFYB	ARNT	PBX1	NR1H2	IRF1	ARNT	NFYB	PBX1	NR1H2	IRF1	ARNT	NFYB
	CREB1	IRF1	AIRE	NR2F2	SRF	SRF	AIRE	IRF1	NR2F2	SRF	SRF	AIRE	IRF1
	NR1H2	IRF7	CD40	CEBPA	NFKB1	ARNT	CD40	IRF7	CEBPA	NFKB1	ARNT	CD40	IRF7
	PBX1	TFDP1	STAT6	STAT1	CD40	CREB1	STAT6	TFDP1	STAT1	CD40	CREB1	STAT6	TFDP1
	ARNT	JUN	RORA		NFYA	JUN	RORA	JUN		NFYA	JUN	RORA	JUN
	CD40	DSP	STAT2		PBX1	POU1F1	STAT2	DSP		PBX1	POU1F1	STAT2	DSP
	GATA1	E2F4	AHR		ARNT	NFE2L1	EGR3	E2F4		ARNT	NFE2L1	EGR3	E2F4
	NFKB1	E2F1	EGR3		NFYB	STAT5A	NFKB1	E2F1		NFYB	STAT5A	NFKB1	E2F1
	NR3C1	PAX6	NFKB1		PGR	RORA	E2F4	PAX6		PGR	RORA	E2F4	PAX6
	TBP	GATA1	E2F4		NR3C1	TP53	STAT5B	GATA1		NR3C1	TP53	STAT5B	GATA1
	CEBPA	CREB1	STAT5B		AR	IL6	TFDP1	CREB1		AR	IL6	TFDP1	CREB1
	AR	PBX1	TFDP1		IRF1	TFAP2A	TBX5	PBX1		IRF1	TFAP2A	TBX5	PBX1
	NFYA	IRF2	TBX5		FOXD1	NFYA	DSP	IRF2		FOXD1	NFYA	DSP	IRF2
	IRF8	FOXO4	DSP		MEIS1	CEBPB		FOXO4		MEIS1	CEBPB		FOXO4
	SRY	MYC			SRY	FOXL1		MAX		SRY	FOXL1		MAX
	IRF2	MAX			CEBPA	ATF2		AIRE		CEBPA	ATF2		AIRE
	STAT3	ZEB1			PAX5	FOS		PPARG		PAX5	FOS		PPARG
	NFYB	AIRE			MAX	EFNA2		E2F3		MAX	EFNA2		E2F3
	HNF4A	PPARG			VDR	STAT4		FOXD1		VDR	STAT4		FOXD1
	AHR	E2F3			PAX6			TBP		PAX6			TBP
	GABPA	FOXD1			REST			SOX9		REST			SOX9
	PAX6	TBP			IRF8			TP53		IRF8			TP53
	PGR	SOX9			JUN			ATF2		JUN			ATF2
	HNF1B	TP53			SOX9			NFKB1		SOX9			NFKB1
	NR2F2	ATF2			FOXJ1			NRF1		FOXJ1			NRF1
	MEIS1	NFKB1			PDX1			FOXC1		PDX1			FOXC1
	JUN	NRF1			STAT3			GATA3		STAT3			GATA3
	PDX1	FOXC1			NR1H3			LMO2		NR1H3			LMO2
	RORA	GATA3			RORA			RXRB		RORA			RXRB
	SOX9	LMO2			IRF2			HNF1B		IRF2			HNF1B

NR1H4	AHR	HNF1B	CEBPB	HNF1B	CEBPB
VDR	RXRB	GATA1	GATA2	GATA1	GATA2
IRF7	HNF1B	FOXC1	ELK1	FOXC1	ELK1
SOX5	CEBPB	FKBP4	HIF1A	FKBP4	HIF1A
FOXF2	GATA2	SOX5	SETD2	SOX5	SETD2
RELA	ELK1	HNF4A	HNF4A	HNF4A	HNF4A
MAX	HIF1A	LEF1	RFX1	LEF1	RFX1
EGR1	SETD2	SREBF1	CD40	SREBF1	CD40
IRF3	HNF4A	CEBPD	FOS	CEBPD	FOS
IRF4	RFX1	ZBTB16	RUNX1	ZBTB16	RUNX1
IRF5	CD40	HOXA9	PDX1	HOXA9	PDX1
LEF1	FOS	ATF6	BACH1	ATF6	BACH1
NR2F1	RUNX1	CNTN2	STAT6	CNTN2	STAT6
POU1F1	PDX1	PLAU	CASR	PLAU	CASR
ARID5B	BACH1	TBP	NR1I2	TBP	NR1I2
CEBPD	STAT6	ONECUT2	FOXF2	ONECUT2	FOXF2
E2F1	CASR	E2F1	POU3F1	E2F1	POU3F1
EGR2	NR1I2	ATF3	MZF1	ATF3	MZF1
PAX5	FOXF2	RREB1	SPIB	RREB1	SPIB
FOS	POU3F1	STAT6	STAT5A	STAT6	STAT5A
ATF6	MZF1	FOXO1	irf3	FOXO1	irf3
FOXA1	SPIB	FOXA1	irf4	FOXA1	irf4
ZBTB16	STAT5A	RFX1	irf5	RFX1	irf5
TP53	IRF3	STAT5B	irf8	STAT5B	irf8
NKX3-1	IRF4	IRF7	stat3	IRF7	stat3
FKBP4	IRF5	RELA	foxj2	RELA	foxj2
FOXO1	IRF8	NR1H4	pou2f2	NR1H4	pou2f2
REST	STAT3	NKX3-1	gata4	NKX3-1	gata4
CNTN2	FOXJ2	NR2F2	nr1h2	NR2F2	nr1h2
MYC	POU2F2	STAT4	bach2	STAT4	bach2
RFX1	GATA4	ATF2	usf2	ATF2	usf2
STAT5B	NR1H2	CASR	arnt	CASR	arnt
ELK4	BACH2	EGR3	atf1	EGR3	atf1
ONECUT1	USF2	NR1I2	gabpa	NR1I2	gabpa
ONECUT2	ARNT	EGR2	AR	EGR2	AR
CEBPB	ATF1	FOXO4	SPI1	FOXO4	SPI1
SREBF1	GABPA	CEBPB	nfe2l1	CEBPB	nfe2l1
FOXO3	AR	NR2F1	cebpg	NR2F1	cebpg
HOXA9	SPI1	ETS1	hsf1	ETS1	hsf1
STAT4	NFE2L1	SP1	nr1h4	SP1	nr1h4
STAT6	CEBPG	FOXO3	ube4a	FOXO3	ube4a
IRF9	HSF1	FOXA2	vdr	FOXA2	vdr
EGR3	NR1H4	FOXA3	NR2F2	FOXA3	NR2F2
NFE2L1	UBE4A	FOXM1	STAT4	FOXM1	STAT4
TEAD1	VDR	FOS	ETS2	FOS	ETS2
ELSPBP1	NR2F2	IRF3	PLAU	IRF3	PLAU
FOXJ1	STAT4	IRF4	GATA6	IRF4	GATA6
FOXA2	ETS2	IRF5	SRY	IRF5	SRY
FOXA3	PLAU	GABPA	SRF	GABPA	SRF
FOXM1	GATA6	RXRB	ESR1	RXRB	ESR1
FOXC1	SRY	ALX1	NR2F1	ALX1	NR2F1
PLAU	SRF	POU1F1	SP3	POU1F1	SP3
ATF2	ESR1	ARID5B	NR1H3	ARID5B	NR1H3
NR1H3	NR2F1	POU3F1	FOXO1	POU3F1	FOXO1
ETS1	SREBF1	SOX10	NHLH1	SOX10	NHLH1
FOXD1	SP3	STAT2	PAX2	STAT2	PAX2
ZEB1	NR1H3	BACH1	RUNX2	BACH1	RUNX2
FOXO4	FOXO1	SP3	TCF3	SP3	TCF3
SOX10	NHLH1	ESR1	ATF3	ESR1	ATF3
FOXL1	PAX2	TP53	GABPB1	TP53	GABPB1
ELF2	RUNX2	SP4	GABPB2	SP4	GABPB2
POU3F1	TCF3	TOPORS	RFX5	TOPORS	RFX5
BACH1	ATF3	TFAP2A	RFXANK	TFAP2A	RFXANK
MYOD1	GABPB1	ZNF589	RFXAP	ZNF589	RFXAP
RREB1	GABPB2	ELSPBP1	FOSL1	ELSPBP1	FOSL1
ALX1	RFX5	ETS2	JUNB	ETS2	JUNB
NR1I2	RFXANK	POU2F2	JUND	POU2F2	JUND
ESR1	RFXAP	SMAD4	TFAP2A	SMAD4	TFAP2A
LHX3	FOSL1	PPARA	TEAD1	PPARA	TEAD1
ETS2	JUNB	DSP	TBX5	DSP	TBX5
ATF3	JUND	FOXD3	GFI1	FOXD3	GFI1
CASR	TFAP2A	IRF9	GFI1B	IRF9	GFI1B
FOSL1	TEAD1	ELF2	CREM	ELF2	CREM
JUNB	TBX5	ATF4	HOXA7	ATF4	HOXA7
JUND	GFI1	ELK1	LEF1	ELK1	LEF1
TFAP2A	GFI1B	MYOD1	FOSL2	MYOD1	FOSL2
USF1	CREM	FOSL1	ARID5B	FOSL1	ARID5B
SP1	HOXA7	JUNB	FOXO3	JUNB	FOXO3
YY1	LEF1	JUND	FOXI1	JUND	FOXI1
DSP	FOSL2	NR6A1	NR5A2	NR6A1	NR5A2
RXRB	ARID5B	ELK4	ATF7	ELK4	ATF7
CREM	FOXO3	USF1	STAT5B	USF1	STAT5B
SMAD4	FOXI1	FOXJ2	LHX3	FOXJ2	LHX3
ALX4	NR5A2	ATF7	ETS1	ATF7	ETS1
FOSL2	ATF7	BACH2	RARA	BACH2	RARA
STAT2	STAT5B	HLF	RARB	HLF	RARB
HLF	LHX3	FOXI1	RARG	FOXI1	RARG
ATF1	ETS1	TFAP4	CEBPD	TFAP4	CEBPD
TCF4	RARA	PAX2	SMAD3	PAX2	SMAD3

FOXD3	RARB
ZNF238	RARG
CEBPG	RXRG
NFIL3	CEBPD
IRF6	SMAD3
BACH2	USF1
SP3	GATA5
PPARA	ATF6
FOXJ2	FOXJ1
POU2F2	ATF4
TFAP4	EFNA2
GATA2	NFATC1
NKX2-1	NFATC2
NKX2-2	NFATC3
NR6A1	NFATC4
AIRE	SOX5
E2F4	BRCA1
ELK1	ETV4
POU2AF1	NKX2-1
POU4F1	FOXL1
POU5F1	NFIL3
POU5F1B	SMAD4
FOXI1	NFIC
NRF1	MEIS1
PAX2	TFE3
TFDP1	TFEB
CUZD1	YY1
FLI1	CEBPE
HSF1	PAX8

FOXL1	USF1
POU2AF1	GATA5
POU4F1	ATF6
POU5F1	FOXJ1
POU5F1B	ATF4
NFKB2	EFNA2
IRF6	NFATC1
SP2	NFATC2
AIRE	NFATC3
E2F4	NFATC4
NFE2L1	SOX5
TFDP1	BRCA1
TCF4	ETV4
NFIC	NKX2-1
ZNF238	FOXL1
PRDM1	NFIL3
EGR4	SMAD4
GATA4	NFIC
FOSL2	MEIS1
MTF1	TFE3
REL	TFEB
	YY1
	CEBPE
	PAX8
	ELF2

FOXL1	USF1
POU2AF1	GATA5
POU4F1	ATF6
POU5F1	FOXJ1
POU5F1B	ATF4
NFKB2	EFNA2
IRF6	NFATC1
SP2	NFATC2
AIRE	NFATC3
E2F4	NFATC4
NFE2L1	SOX5
TFDP1	BRCA1
TCF4	ETV4
NFIC	NKX2-1
ZNF238	FOXL1
PRDM1	NFIL3
EGR4	SMAD4
GATA4	NFIC
FOSL2	MEIS1
MTF1	TFE3
REL	TFEB
	YY1
	CEBPE
	PAX8
	ELF2

Supplemental data 6

regulation of the Warburg effect

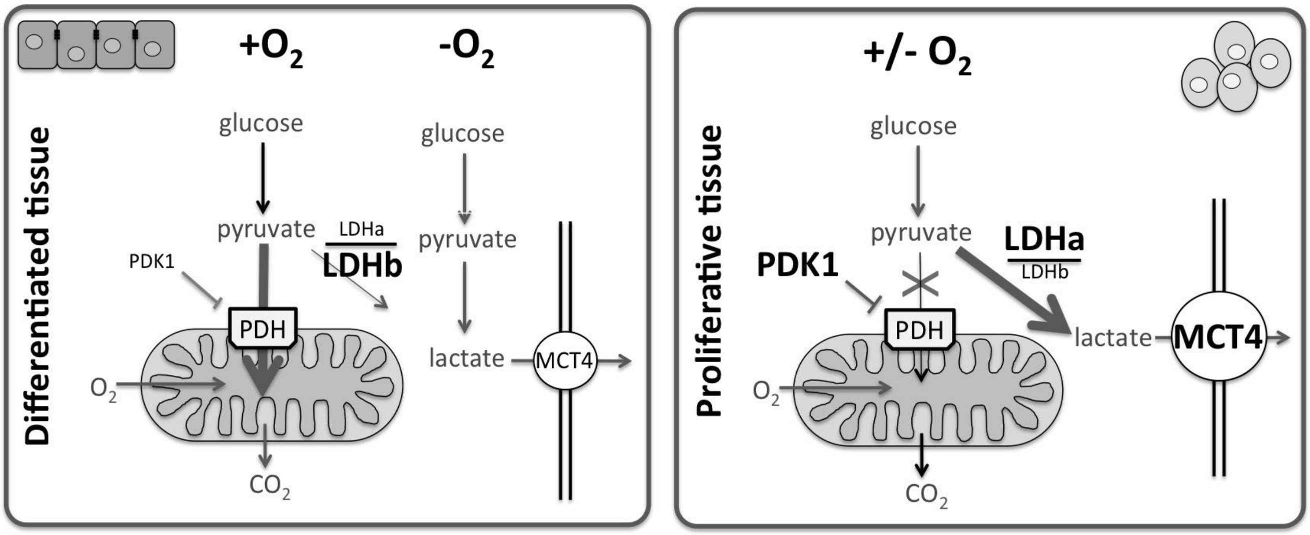

Proliferating cells often display a metabolic profile that is referred to as 'cancer metabolism', first described by Otto Warburg in 1924. Cancer metabolism is typically characterized by reduced levels of oxidative phosphorylation and mitochondrial activity, higher dependence on aerobic glycolysis and glutaminolysis for ATP production, and increased generation of biosynthetic intermediates that are essential for the production of macromolecules (phospholipids, nucleotides, proteins) to support cell proliferation (reviewed in[3, 5-7]). The metabolic reprogamming that occurs during the Warburg effect is tightly regulated. The likely benefit for proliferative cells to adopt a Warburg-like metabolic profile, despite the much lower yield of ATP, is the conservation of pyruvate for the synthesis of lipids, nucleotides and amino acids, as building blocks for new cells [1-4].

Key regulators of the Warburg effect include:

* **PDK1** (pyruvate dehydrogenase kinase 1): in proliferative cells PDK1 is up-regulated, inhibiting PDH (pyruvate dehydrogenase), which transport pyruvate into the mitochondria, PDK1 upregualtion results in limiting the uptake of pyruvate into the mitochondria. *PDK1 is down-regulated in HS-cultured cells, in line with a non-proliferative character. PDH did not change.*

*** LDHA and LDHB:** lactate dehydrogenases A and B catalyze the conversion of pyruvate ot lactate (LDHA), vice versa (LDHB) High LDHA levels direct the conversion of pyruvate to lactate, whereas low LDHa levels, with higher LDHB levels favour the reverse reaction. *LDHA is decreased in HS cultured cells, whereas LDHB is increased, consistent with the ratio in differentiated tissue*

*** MCT4** (monocarboxylic acid transporter 4): MCT4 removes lactate from the cells, and is increased during aerobic glycolysis (right panel). *MCT4 is decreased in HS cultured cells, consistent with the reversal of the Warburg effect.*

1. Cairns, R.A., Harris, I.S. & Mak, T.W. Regulation of cancer cell metabolism. Nat Rev Cancer 11, 85-95 (2011).
2. Lunt, S.Y. & Vander Heiden, M.G. Aerobic glycolysis: meeting the metabolic requirements of cell proliferation. Annu Rev Cell Dev Biol 27, 441-464 (2011).
3. Pavlova, N.N. & Thompson, C.B. The Emerging Hallmarks of Cancer Metabolism. Cell Metab 23, 27-47 (2016).
4. Vander Heiden, M.G., Cantley, L.C. & Thompson, C.B. Understanding the Warburg effect: the metabolic requirements of cell proliferation. Science 324, 1029-1033 (2009).

Supplemental Data 7: 25 genes with highest increase or decrease in expression

Increased expression (Top 25)

	Gene Title	Gene Symbol	fold increase d8	d15	d23
1	sulfotransferase family 1E, estrogen-preferring, member 1	SULT1E1	1.60980851	10.074521	**17.2660059**
2	apolipoprotein A-IV	APOA4	1.46445382	3.96256758	**12.9515673**
3	urothelial cancer associated 1 (non-protein coding)	UCA1	13.0403278	14.1624532	**12.653981**
4	S100 calcium binding protein A14	S100A14	1.10835538	2.47252987	**12.1360695**
5	anterior gradient 2 homolog (Xenopus laevis)	AGR2	4.09387803	7.3550794	**11.4152296**
6	phospholipase A1 member A	PLA1A	1.72761844	6.24060598	**11.0728783**
7	Kruppel-like factor 4 (gut)	KLF4	4.8156499	6.25374168	**10.9723147**
8	epithelial splicing regulatory protein 1	ESRP1	1.72298527	4.746049	**10.2605455**
9	guanylate binding protein 2, interferon-inducible	GBP2	4.90674414	7.71908765	**10.2079568**
10	transcription factor EC	TFEC	2.9324839	5.22814844	**9.95592446**
11	leucine rich repeat containing 19	LRRC19	1.10196646	2.89413995	**9.76210338**
12	zinc finger protein 114	ZNF114	2.96517484	4.5009058	**9.35384665**
13	cholinergic receptor, nicotinic, alpha 1 (muscle)	CHRNA1	3.89463693	4.89524151	**8.91442928**
14	discoidin domain receptor tyrosine kinase 1 /// microRNA 4640	DDR1	1.01238471	3.17424196	**8.03071659**
15	chromosome 19 open reading frame 69	C19orf69	1.08588648	2.76599503	**7.98168685**
16	UDP glucuronosyltransferase 2 family, polypeptide A3	UGT2A3	1.85090717	6.82158978	**7.87140438**
17	protease, serine, 23	PRSS23	3.95576075	6.46029912	**7.76669156**
18	chromosome 12 open reading frame 39	C12orf39	4.53196492	6.43655253	**7.71021042**
19	proline/histidine/glycine-rich 1	PHGR1	1.80191957	5.29692291	**7.02058702**
20	anoctamin 1, calcium activated chloride channel	ANO1	0.97804907	0.94345729	**6.98110729**
21	vanin 2	VNN2	11.4370228	10.9074657	**6.93981919**
22	insulin-like growth factor binding protein 3	IGFBP3	0.93963018	2.63802643	**6.76395235**
23	hephaestin	HEPH	2.6201261	4.21156181	**6.52502213**
24	cell death-inducing DFFA-like effector c	CIDEC	4.24499095	4.38011981	**6.48955863**
25	family with sequence similarity 47, member E	FAM47E	4.80857272	7.66127981	**6.25362451**

Decreased expression (Top 25)

	Gene Title	Gene Symbol	fold decrease d8	d15	d23
1	fibrinogen beta chain	FGB	1.01799746	2.61994629	**5.23852945**
2	leukemia inhibitory factor	LIF	5.24506451	5.02385697	**4.77031089**
3	tubulin, beta 1 class VI	TUBB1	2.94554978	3.25901429	**4.70925991**
4	glucan (1,4-alpha-), branching enzyme 1	GBE1	2.20827505	3.07756181	**4.11316342**
5	coagulation factor XIII, B polypeptide	F13B	2.234701	3.08244714	**3.96939829**
6	reelin	RELN	2.70351405	2.92224273	**4.83406186**
7	alcohol dehydrogenase 6 (class V)	ADH6	3.36367076	3.17448993	**4.49200411**
8	protein phosphatase 1, regulatory (inhibitor) subunit 1A	PPP1R1A	3.86675208	3.40475789	**3.49474836**
9	cancer susceptibility candidate 5	CASC5	2.32440917	2.14682899	**3.47556406**
10	F-box and leucine-rich repeat protein 21 (gene/pseudogene)	FBXL21	2.56581742	1.99000289	**3.40485515**
11	forkhead box N4	FOXN4	2.82919142	3.00748786	**3.30598489**
12	natriuretic peptide B	NPPB	0.73137249	1.77084611	**3.2991722**
13	claudin 14	CLDN14	3.44809761	3.16915066	**3.28154663**
14	fucosyltransferase 11 (alpha (1,3) fucosyltransferase)	FUT11	1.55861651	2.67772711	**3.17790018**
15	ribonucleotide reductase M2	RRM2	1.22693238	1.67626212	**3.57449496**
16	SPC25, NDC80 kinetochore complex component, homolog (S. cerevisiae)	SPC25	1.69842724	1.92727121	**3.13577253**
17	E2F transcription factor 7	E2F7	2.29203247	2.27124832	**3.05634284**
18	alanine-glyoxylate aminotransferase 2-like 1	AGXT2L1	1.11519966	1.67205374	**3.40744429**
19	fibrinogen alpha chain	FGA	2.0769312	3.00503011	**3.4734301**
20	6-phosphofructo-2-kinase/fructose-2,6-biphosphatase 4	PFKFB4	3.22650034	3.78462628	**3.42101327**
21	pyruvate dehydrogenase kinase, isozyme 1	PDK1	2.32540542	2.45489876	**3.07676876**
22	non-SMC condensin I complex, subunit G	NCAPG	1.98587391	2.51523109	**3.02975911**
23	hypoxia inducible lipid droplet-associated	HILPDA	1.63404113	2.40863783	**3.15806134**
24	kinesin family member 14	KIF14	1.26819019	1.80179264	**3.40913024**
25	zinc finger protein 789	ZNF789	1.45259678	2.17329004	**2.86947321**

Supplemental Data 9

mRNA levels determined by quantitative PCR of different cytochrome P450 genes.

A: CYP1, 2 and 3 families: drug and steroid metabolism

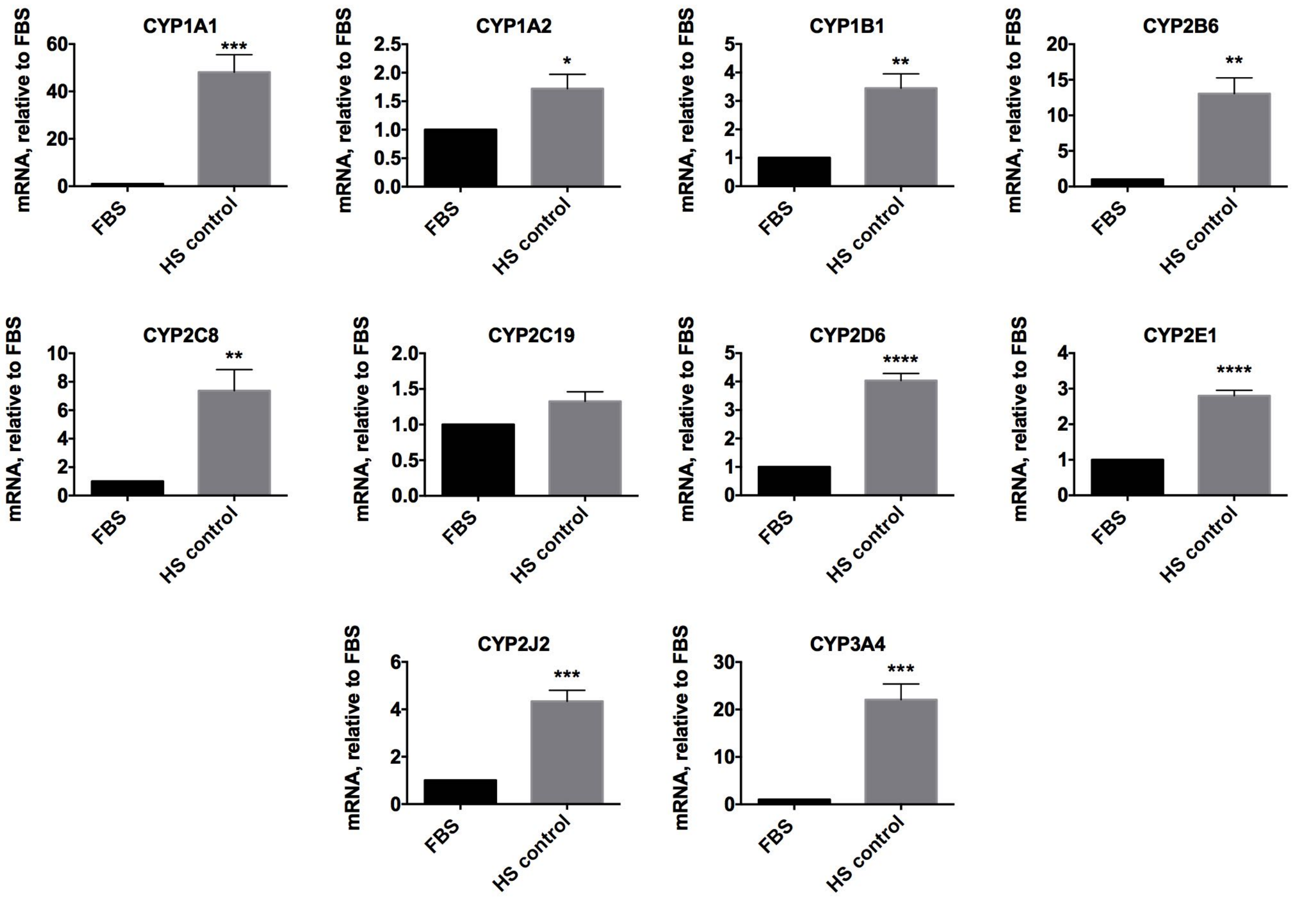

B: CYP4 familly: arachidonic acid or fatty acid metabolism

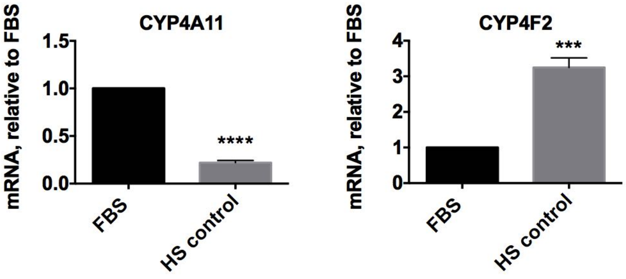

mRNA levels determined by quantitative PCR of other factors involved in degradation or removal of xenobiotics

Epoxide hydrolases:

detoxifiction of exogenous chemicals such as polycyclic aromatic hydrocarbons

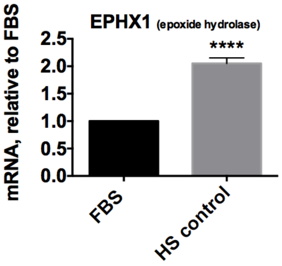

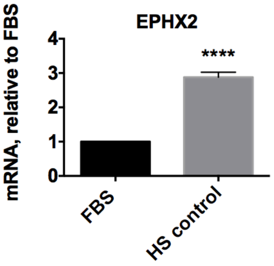

NQO1, GSTA1:

removal of lipid peroxidation by-products, carcinogens, bilirubin

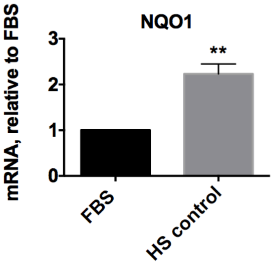

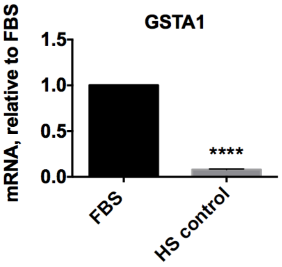

UDP-glucoronosulfotransferases:

transforms lipophilic molecules such as steroids, bilirubin, hormones, analgesic and other drugs to water soluable compounds

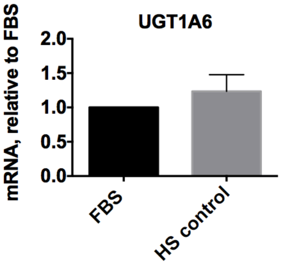

ABC transporters:

transport of a wide variety of substrates out of the cell, including metabolic products, lipids, steroids, and drugs. ABCA1 removes excess cholesterol from the cell, ABCB1 is also known as MDR-1, ABCC1 is also known as MRP1, ABCD1 transports fatty acids into the peroxisome,

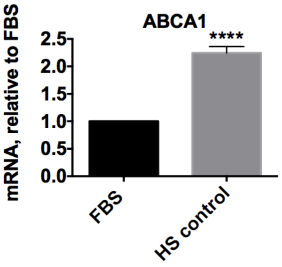

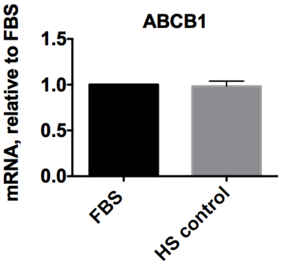

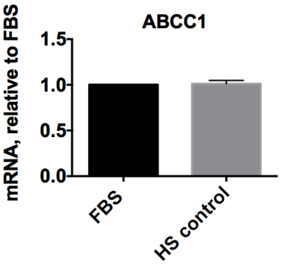

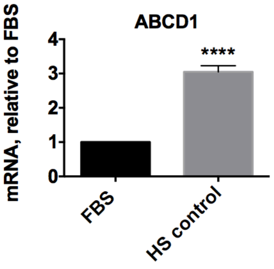

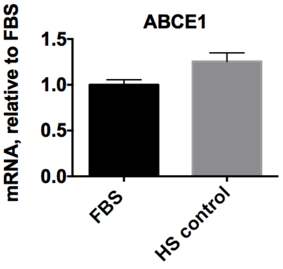

Supplemental data 10

Full length blot of figure 5C

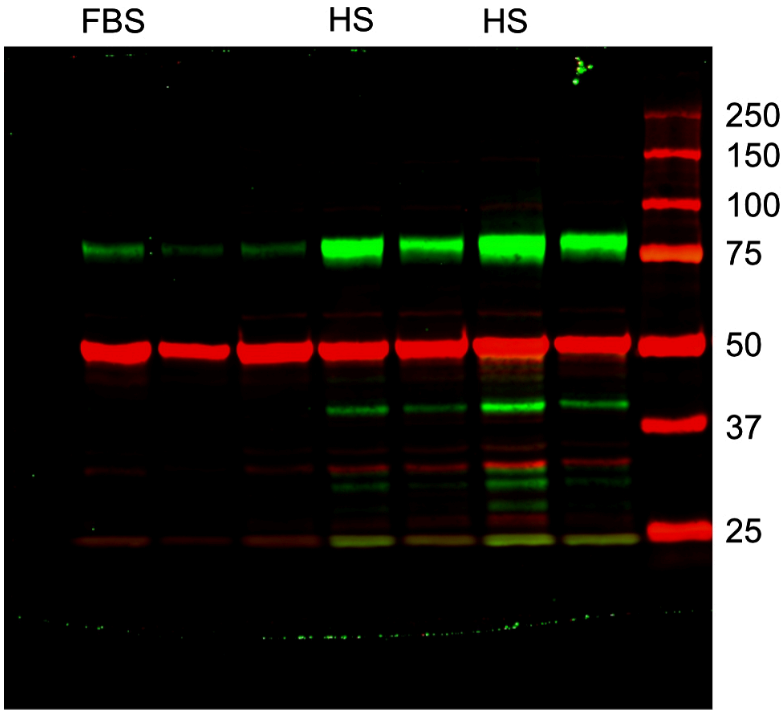

Full length Licor blot of the cropped images of figure 5C. CPT-1 has a predicted size of 78 kDa and is stained green. Tubulin and the molecular weight markers are stained red. Additional, unlabeled lanes on the blot represent other conditions that are not relevant for this study.
